# High-resolution Repli-Seq defines the temporal choreography of initiation, elongation and termination of replication in mammalian cells

**DOI:** 10.1186/s13059-020-01983-8

**Published:** 2020-03-24

**Authors:** Peiyao A. Zhao, Takayo Sasaki, David M. Gilbert

**Affiliations:** Department of Biological Science, Florida State University, 319 Stadium Drive, Tallahassee, FL 32306 USA

## Abstract

**Background:**

DNA replication in mammalian cells occurs in a defined temporal order during S phase, known as the replication timing (RT) programme. Replication timing is developmentally regulated and correlated with chromatin conformation and local transcriptional potential. Here, we present RT profiles of unprecedented temporal resolution in two human embryonic stem cell lines, human colon carcinoma line HCT116, and mouse embryonic stem cells and their neural progenitor derivatives.

**Results:**

Fine temporal windows revealed a remarkable degree of cell-to-cell conservation in RT, particularly at the very beginning and ends of S phase, and identified 5 temporal patterns of replication in all cell types, consistent with varying degrees of initiation efficiency. Zones of replication initiation (IZs) were detected throughout S phase and interacted in 3D space preferentially with other IZs of similar firing time. Temporal transition regions were resolved into segments of uni-directional replication punctuated at specific sites by small, inefficient IZs. Sites of convergent replication were divided into sites of termination or large constant timing regions consisting of many synchronous IZs in tandem. Developmental transitions in RT occured mainly by activating or inactivating individual IZs or occasionally by altering IZ firing time, demonstrating that IZs, rather than individual origins, are the units of developmental regulation. Finally, haplotype phasing revealed numerous regions of allele-specific and allele-independent asynchronous replication. Allele-independent asynchronous replication was correlated with the presence of previously mapped common fragile sites.

**Conclusions:**

Altogether, these data provide a detailed temporal choreography of DNA replication in mammalian cells.

## Introduction

DNA replication in eukaryotes proceeds in a defined temporal order known as the replication timing (RT) programme [1]. RT is dynamically regulated during development and exhibits cell-type-specific RT signatures [2, 3]. RT is also closely correlated with the A/B compartment of chromatin structure, the local chromatin environment and the transcription potential of the region [4–7]. One of the primary assays for genome-wide RT in mammalian cells has been E/L Repli-Seq in which cells labelled with BrdU for 10–20% of S phase are sorted into early and late S fractions and RT profiles are generated from the log2 ratio of read enrichment in the BrdU-immunoprecipitated early fraction to that in the late fraction (E/L) [8]. Previous studies have also reported multi-fraction Repli-Seq approaches using 4–6 fractions or stages of S phase [9–11] or have generated replication timing profiles from the copy number of sequences in proliferating cell populations [12].

RT profiles reveal large constant timing regions (CTRs) of early and late replication manifesting as plateaus and punctuated by timing transition regions (TTRs) of rightward or leftward slopes [13]. Early and late CTRs must contain sites of replication initiation as they replicate too rapidly to be accounted for by elongation alone. By contrast, TTRs are hypothesised to consist mainly of uni-directional forks, occasionally accelerated by origin firing [2, 14, 15]. However, measurements supporting these hypotheses derive from prior RT profiling methods that smoothed data over hundreds of kilobases (kbs) and/or relied on long metabolic labels incorporated over large stretches (> 200 kb) of DNA and so lacked the resolution to identify sites of replication initiation within CTRs and TTRs. Moreover, since these methods average all stochastic variation in a cell population, they do not permit one to determine the degree of cell-to-cell RT variation. We previously developed a single-cell Repli-Seq method to address this problem, concluding that the RT programme is stable from cell to cell, but this method suffers from low resolution due to the limited breadth of whole genome single-cell sequencing and the single temporal snapshot obtained from each cell [16, 17].

Mammalian replication origin mapping poses a significant challenge due to the high flexibility of sites that can initiate replication and their varying efficiencies [18, 19]. Moreover, frequently, clusters of origins used at varying efficiencies produce what have been called ‘initiation zones’ (IZs). Small nascent strand sequencing (SNS-seq) and Okazaki fragment sequencing (OK-seq) methods have produced comprehensive maps of replication origins and fork polarities, respectively, in several human and mouse cell lines [20–24]. SNS can map sites to kb resolution, but the sites detected must fire frequently enough to detect above noise; clusters of inefficient origins escape detection. OK-seq detects transitions in fork polarity that defines bi-directional replication to kb resolution in yeast [25], but in mammalian cells, these transitions are more gradual, consistent with the prevalence of IZs. Mapping origins on single DNA molecules can, in principle, measure the frequency of initiation at specific sites, but existing methods are extremely low throughput. Studies analysing hundreds of DNA fibres from a single genomic location have revealed that some regions initiate at defined sites while other genomic locations can initiate at many sites distributed broadly, with each site used in less than 2% of S phases [26–28]. By contrast, RT shows little cell-to-cell variation [16, 17]. Thus, the prevailing view is that a deterministic RT programme emerges from stochastic origin selection [29]. However, there is a need for more sensitive methods to map the landscape of origin and fork distributions genome-wide in mammalian cell.

To address these gaps, we developed an approach that can delineate IZs, TTRs and termination sites with unprecedented temporal resolution in 3 human cell lines and 2 mouse cell types. We identify specific sites of replication initiation activity within TTRs and resolve them from stretches of uni-directional replication or true TTRs. We detect a remarkable homogeneity in the temporal order of replication in cell populations, at considerably higher resolution than achieved by our prior single-cell measurements [16]. Whereas active histone modifications were consistently correlated with early firing IZs, repressive histone marks varied between cell types in their relationship with IZ initiation time. However, initiation time was intimately linked to Hi-C compartment and early IZs were enriched at Hi-C (insulation score defined) domain boundaries. We show that developmental transitions are regulated primarily by turning on or off initiation activity within single IZs, suggesting that the multiple initiation sites within an IZ are regulated as a unit. The temporal resolution permitted the identification of biphasically replicated regions and the extensive read depth and breadth permitted haplotype phasing, which revealed both allele-specific replication asynchrony and allele-independent asynchrony, the latter of which was associated with long transcribed genes and common fragile sites (CFS).

## Results

### 16 fraction Repli-seq reveals patterns of replication with high temporal resolution

**Fig. 1:**
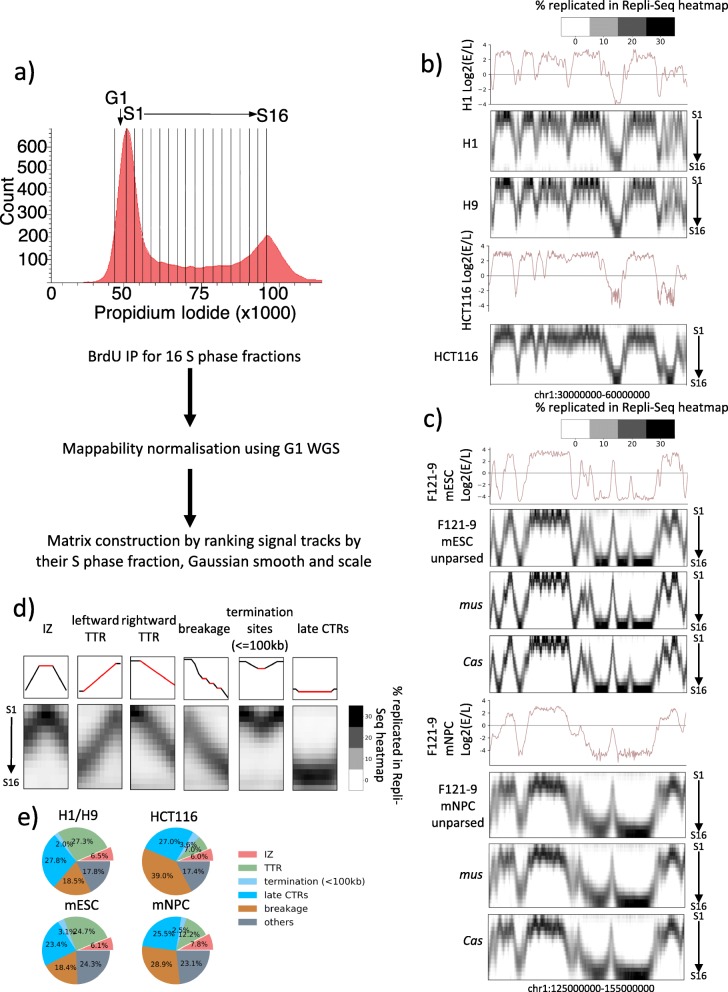
High-resolution Repli-Seq produces robust and reproducible heatmaps that annotate features of replication at fine temporal resolution. **a** Experimental and analysis flow for high-resolution Repli-seq. **b** E/L Repli-seq for H1 hESCs (top), normalised High-Res Repli-seq heatmaps for chr1:30,000,000–60,000,000 in H1, H9 and HCT116. **c** E/L Repli-seq for F121-9 mESCs and mNPCs, normalised High-Res Repli-seq heatmaps for chr1:125,000,000–155,000,000 in unparsed *mus* allele and *cas* allele in mESC and mNPC. **d** 5 Features observed in high-resolution Repli-Seq heatmap. Top row: line plots showing the rank of the cluster centroid with which the current bin is associated (red indicates the bins of interest that constitute the corresponding feature and black indicates the surrounding bins). Bottom row: corresponding heatmaps of IZs, leftward TTR, rightward TTR, breakages in TTR, short termination sites (≤ 100 kb) and late CTRs. **e** Percentage of genome constituted by features in **d**

We performed high-resolution Repli-Seq in 5 cell types: three human cell lines, male and female human embryonic stem cell (hESC) lines H1 (WA01) and H9 (WA09) hESCs and human colorectal cancer line HCT116 as well as mouse embryonic stem cell (mESC) line F121-9 derived from hybrid *castaneusXmusculus* mouse embryos and finally neural progenitor cells (mNPCs) derived from F121-9 (‘Materials and methods’ section, Additional file 1: Fig. S1). Cells were labelled with BrdU for 30 min, stained with propidium iodide and sorted by FACS into 16 equal S phase fractions (Fig. 1a). Sixteen fractions were chosen due to their approximate equivalence to the fraction of S phase labelled with BrdU (30 min of an 8–10-h S phase) as well as convenience with 4-way FACS sorting. BrdU-immunoprecipitated DNA from each fraction was validated by qPCR on HBA (alpha-globin) and HBB (beta-globin) whose RT was known from prior E/L Repli-Seq data and validated in high-resolution Repli-Seq (Additional file 1: Fig. S2a,b). BrdU pull-down efficiency was measured using spike-in to ensure consistency between S phase fractions (‘Materials and methods’ section, Additional file 1: Fig. S3a). The raw read counts per 50-kb bin for each S phase fraction were corrected for mappability using G1 whole genome sequencing (Additional file 1: Fig. S3b,c). To ensure that the G1 control did not contain replicated DNA from early S-phase cell contamination, we performed BrdU immunoprecipitation of DNA purified from G1 phase cells (the ‘Materials and methods’ section) and found no evidence of specific BrdU incorporation (Additional file 1: Fig. S4). We plotted the ranges of log2 (E/L) RT associated with bins that were filtered out as a result of mappability normalisation and found that the RT values of filtered bins excluded those that were representative of the corresponding S phase fraction (Additional file 1: Fig. S3d), confirming that mappability correction removes noise without compromising signal. Furthermore, Repli-Seq heatmaps of corrected mappability were visually inspected (Additional file 1: Fig. S5a) to ensure that features such as diffused peaks in the heatmap, which represent less efficient IZs, were preserved. Datasets were subsequently Gaussian smoothed and normalised between fractions by scaling column-wise between bins so that the sum for any individual column amounted to 100 (‘Materials and methods’ section, Fig. 1a, Additional file 1: Fig. S5b). High-resolution Repli-Seq heatmaps show high concordance with E/L Repli-Seq while providing insights into the finer features of replication that we will further expound in the next section (Fig. 1b–d). After normalisation and scaling, there occasionally could be seen residual noise manifesting as faint pixels in late S phase fractions for very early replicating bins (see Fig. 1b H1 Repli-Seq heatmap) or in early S phase fractions for very late replicating bins (see Fig. 1c unparsed F121-9 mESC Repli-Seq heatmap). We plotted the normalised read distributions in 16 S phase fractions for top 10% earliest replicating regions and 10% latest replicating regions defined using E/L Repli-Seq datasets and determined that this source of noise accounts for < 2% replication for all cell lines studied (Additional file 1: Fig. S6). We speculate that this noise could result from non-specific BrdU pulldown at extreme ends of S phase. It is unlikely to be biological because it is not genome-wide and not present in every dataset. Comparing our H1 data to H1 hESC datasets from prior 6-stage Repli-seq [9] that were normalised and scaled in the same manner, we found that high-resolution Repli-Seq captured all replication features present in datasets from [9] with high correlation (Additional file 1: Fig. S7a, b). However, the considerably higher temporal resolution of the datasets in this work allowed the identification of features that previously escaped detection such as diffused peaks and biphasically replicated regions (indicated by black arrows) (Additional file 1: Fig. S7a). Consistent with the prior knowledge that RT profiles are cell type specific, we find high genome-wide similarity between H1 and H9 16 fraction datasets while the HCT116 dataset shows less correlation with hESCs (Additional file 1: Fig.S8). Mouse heatmaps are generated for both maternal *musculus* (*mus*) and paternal *castaneous* (*cas*) genomes after allele parsing. The two genomes in mESCs and those in mNPCs show high correlation within the same cell type and lower correlation across cell types (Additional file 1: Fig. S8).

#### Defining features in Repli-seq heatmaps: IZs, TTRs, TTR breakages, termination sites and late CTRs

Using high-resolution Repli-Seq, we were able to identify 5 distinct features in the landscape of DNA replication across the genome (Fig. 1d). We first clustered the normalised and scaled Repli-Seq arrays by applying BIRCH algorithm [30] to assign bins to clusters characterised by cluster centroids, which were first sorted according to the fraction where maximum replication occurs from early to late then sorted according to the magnitude of replication in the fraction where maximum replication occurred (Additional file 1: Fig. S9a). We subsequently generated the cluster centroid rank profile for all bins (top row in Additional file 1: Fig. S9b) with earlier replicating bins being assigned to clusters with earlier centroids (left end of Additional file 1: Fig. S9a) and later ones being assigned to clusters with increasingly later centroids (right end of Additional file 1: Fig. S9a). Since origins are replicated earlier than their surrounding regions, and since the window size is 50 kb and cannot distinguish single highly efficient origins from < 50-kb clusters of less efficient origins, we defined ‘initiation zones’ (IZs) as sites manifesting as vertical peaks in the centroid profile. Emanating from IZs are segments of constant slope traversing through several S phase temporal windows, which represent the TTRs, presumably consisting of replication forks moving away from initiation sites during the progression of S phase. Depending on the directionality of progression of the forks emerging from IZs, in Fig. 1d, TTRs are illustrated as either leftward or rightward TTRs (ascending or descending) but are considered a single feature of the data. Frequently found flanked by TTRs are small decreases in slope (hereafter referred to as ‘breakages’) that had escaped detection in prior lower resolution datasets. These regions likely signify origin firing accelerating the rate of DNA replication at these regions. Breakage bins were defined as continuous bins assigned to the same cluster centroid whose neighbouring bins on one end were assigned to earlier replicating cluster centroids while neighbouring bins on the other end were assigned to later replicating cluster centroids. Therefore, breakages were flanked by TTRs on both sides yet were not called as part of TTRs. V-shaped features represent defined sites of replication termination (< 100 kb) where opposing replication forks fuse. Finally, large U-shaped late CTRs are too large and synchronously replicated to be explained by fork fusion and therefore must contain replication origins [31]. While IZs constitute less than 10% of the genome (Fig. 1e), more than half of the genome consists of breakages or late CTRs, indicating that the majority of the genome has detectable replication initiation potential. Origin-free regions, which constitute TTRs and termination sites (< 100 kb), constitute ~ 15–30% of the genome. Altogether, these results provide a comprehensive view of the kinetics of DNA replication genome-wide conserved in several mammalian cell types.

**Fig. 2:**
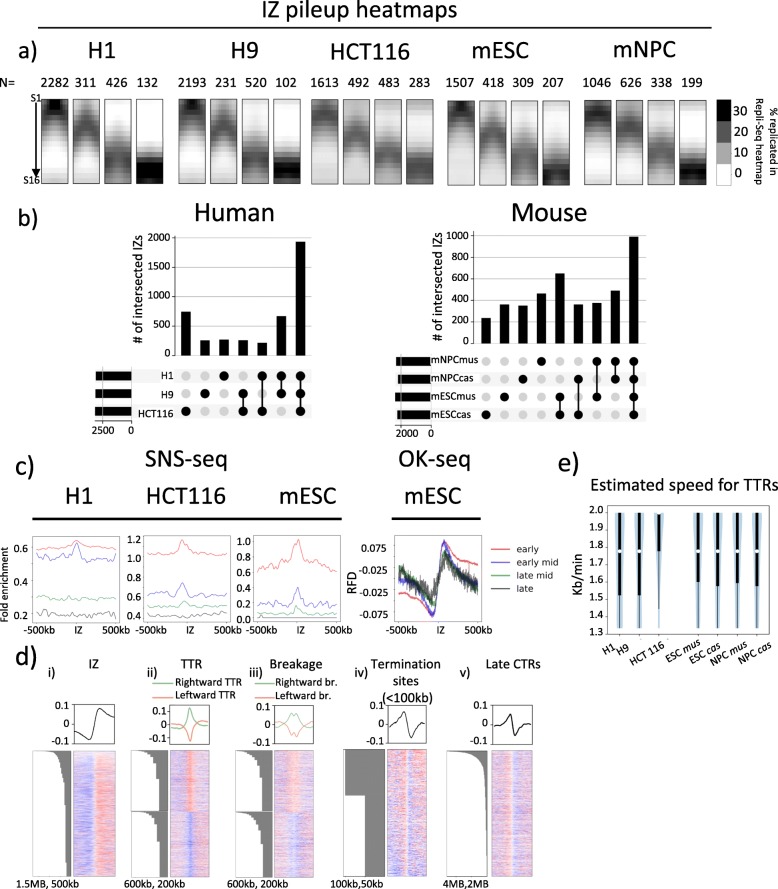
High-resolution Repli-Seq identifies features highly concordant with Ok-seq. **a** Pile-ups of IZs that are categorised into four categories: early, early-mid, late-mid and late by the timing of initiation in H1 and H9 hESCs, HCT116, F121-9 mESCs and mNPCs. The number of IZs in each category is shown on top of the pile-up heatmap. **b** UpSet plots showing numbers of IZs either unique to each cell type alone or uniquely in common between the connected cell types in human and mouse cell lines. Black bars on the lower left are total IZs in each cell type. **c** Mean line plots showing SNS-seq signal (for H9, HCT116 and mESCs) and OK-seq signal (for mESCs) centred at IZs of early (red), early-mid (blue), late-mid (green) and late (black) RT ± 500 kb. **d** Mean line plots (top row) and heatmaps (bottom row) showing mESC OK-seq signal centred at centres of IZs (i), TTRs (ii), breakages (iii), termination sites (< 100 kb) (iv) and termination sites (> 100 kb) (v) ± 1 MB from feature centre. Negative values indicate an enrichment for Okazaki fragments at leftward fork and positive values indicate an enrichment for Okazaki fragments at rightward fork. Each row in a heatmap represents a single site of any feature. Heatmaps are sorted by row using site size. Barplots indicating feature size associated with the corresponding row are on the left of the heatmap. TTR (ii) and breakage (iii) heatmaps are arranged so that rightward TTR/breakages are stacked on top of leftward TTR/breakage and sorted separately. Green and orange mean line plots represent the rightward and left leftward TTRs, respectively. **e** Distribution of estimated TTR speed in kb/min for H1, H9, HCT116, mESC and mNPC. White dots represent the medians of distribution. Thick black lines mark the distribution between the first quartile to the third quartile. Thin black lines indicate the complete distribution of TTR speeds

We identified ~ 3000 IZs in human cell lines and ~ 2400 IZ in mouse cell lines. We classified IZs into early (S1–3), early-mid (S4–6), late-mid (S7–9) and late S (S10–12) IZs depending on the S phase fractions where the highest read density was identified (see the ‘Materials and methods’ section). Pile-up heatmap images centred on each of these temporally defined IZs in each cell type are shown in Fig. 2a. In both human and mouse, we identified constitutive IZs that are shared between cell types as well as cell-type-specific IZs (Fig. 2b). A significant proportion of IZs are shared between cell types (1933 in human cell lines and 991 in mouse for IZs shared between cell types as well as between alleles). H1 and H9 share an intersecting set of 670 IZs that are unique to hESCs while 746 IZs are unique to HCT116. mESC *mus* and *cas* alleles share 650 mESC-specific IZs while mNPC *mus* and *cas* alleles share 464 mNPC-specific IZs.

Next, we aligned our IZs with IZs and initiation sites identified through other origin mapping methods applied in these cell lines, namely small nascent strand sequencing (SNS-seq) [22–24] and Okazaki fragment sequencing (OK-seq) [21]. For H9, HCT116 and the *musculus* genome in mESCs, the SNS-seq shows an enrichment of SNS signal centred around early IZs but not late IZs (Fig. 2c). Moreover, the magnitude of SNS-seq signal within and surrounding the IZs decreases as the timing of initiation within IZs becomes later in S phase. The trend was true across all human and mouse cell types examined, although quantitative cell-type-specific differences exist. On the other hand, IZs show the expected transition in fork polarity that signifies the presence of initiation sites in Ok-seq dataset of the same cell type (mESCs) for all IZs regardless of their S phase initiation time (Fig. 2 c and d (i)). Even the very late IZs that are devoid of SNS-seq origin signal correspond to regions of OK-seq polarity transition showing that OK-seq and high-resolution Repli-Seq validate each other and identify IZs of the same dynamic characteristics. Reciprocally by centring on all called OK-seq IZs [21] and plotting the pile-up of high-resolution Repli-Seq signal, we show that *called* OK-seq IZs are primarily early replicating (Additional file 1: Fig. S10a,b). mESC OK-seq identified 2844 IZs while high-resolution Repli-Seq identified 2441 IZs. We further identified IZs that are unique to OK-seq (*n* = 850) and Repli-seq (*n* = 604) (Additional file 1: Fig. S10c). Out of 850 OK-seq unique IZs, 667 overlapped with Repli-Seq TTR breakages that were not called as IZs but are consistent with the presence of initiation activities within these regions. The rest were either embedded in large late CTRs (Additional file 1: Fig. S10cii,d), still consistent with Repli-seq IZs, or simply did not align, being found within TTRs or termination sites (< 100 kb) in Repli-Seq heatmaps. Indeed, the OKseq unique IZs closer to Repli-Seq IZs tended to be earlier replicating and those that were at a greater distance to Repli-Seq IZs were later replicating (Additional file 1: Fig. S10e); we speculate that these may be false positives. On the other hand, Repli-Seq unique IZs were associated with an ascending gradient of OK-seq replication fork directionality (RFD), consistent with the presence of initiation activity but the magnitude of ascension was small (Additional file 1: Fig. S10f) potentially accounting for why these RFD transitions were not called as OK-seq IZs. Overall, we conclude that the SNS method is primarily able to detect early replicating origins, while OK-seq detects polarity shifts at every temporal transition in the data.

Due to the high consistency between Repli-Seq IZs and OK-seq signal, we examined OK-seq signal centred around all of the dynamic features of replication described in Fig. 1d. TTRs were divided into either rightward or leftward TTRs and associated with positive or negative OK-seq signal values, respectively, consistent with uni-directional fork movement (Fig. 2d (ii)). In addition, SNS-seq signal around rightward or leftward TTRs aligned at TTR centres revealed a sharp drop over the span of ~ 50 kb (equivalent of one genomic bin) in signal enrichment, indicating that TTRs were indeed depleted of SNS-detected origins and segregated IZs that were rich in efficient origins from termination sites that contained either no origins or inefficient origins (Additional file 1: Fig. S11). TTR breakages and late CTRs are expected to contain origin activity to replicate so rapidly (albeit small ones could, in principle, be regions of unusually rapid fork movement). In fact, TTR breakages were detected as a drop in fork polarity in either rightward or leftward fork movement depending on the directionality of the TTR in which the breakage is located, consistent with origin activity causing diminished polarity bias of Okazaki fragments within TTRs (Fig. 2d (iii)). The presence of breakages shows that TTRs as previously defined contain origin activity, thus contributing to variation in smoothed TTR slope gradients. As expected, termination sites, regardless of their size, show red to blue (positive to negative) transition in OK-seq that represents the fusion of opposing replication forks in termination zones (Fig. 2d (iv), Fig. 2d (v)). While smaller termination sites (< 100 kb) may be devoid of origin activity, late CTRs must contain origins to replicate so rapidly. Consistently, polarity transitions at late CTRs are of a lower magnitude overall than those at smaller termination sites (< 100 kb) (compare the maxima and minima between line plots at the top of Fig. 2d (iv) and Fig. 2d (v), which represent the degrees of uniformity in terms of fork polarity of leftward and rightward forks, respectively), suggesting the presence of increased origin activity that diminishes the polarity bias of Okazaki fragments. In addition, the fact that polarity transitions at termination sites (red to blue) are less uniform than those at IZs (blue to red) suggests that termination is more heterogeneous than initiation.

#### TTRs are highly uniform and consistent with long uni-directional forks

Our demonstration that TTR breakages represent small IZs that disrupt TTR slope implies that removing breakages from TTR analysis should enable more accurate estimation of fork speed within TTRs. In fact, we found that such ‘breakage-free’ TTRs are remarkably uniform in slope and defined in replication timing within the population. In order to estimate the frequency of active origin firing within TTRs, we calculated the distribution of fork speed inferred by dividing TTR sizes by fractions of S phase traversed by TTRs in human and mouse cell lines. Assuming a 10-h S phase (the maximum for the 5 cell lines profiled), the median speed of fork progression for both human and mouse was between 1.7 and 1.8 kb/min (with the exception of HCT116, which has a median of 2 kb/min) and agrees with the range of estimated fork speed measured directly by DNA fibre methods by others (Fig. 2e) [32, 33]. Although we cannot rule out the possibility of sequential origin firing with inter-origin distances < 50 kb (our limit of detection), there would need to be a nearly uniform density and firing efficiency of origins along the length of each TTR to escape detection. Neither OK-seq nor the SNS data nor single DNA fibre mapping data [28] support such an initiation pattern in any of the cell lines analysed. We conclude that TTRs are remarkably uniform in replication speed within a population and can be accounted for by uni-directional fork movements devoid of origin firing.

#### Measuring RT heterogeneity genome-wide

**Fig. 3:**
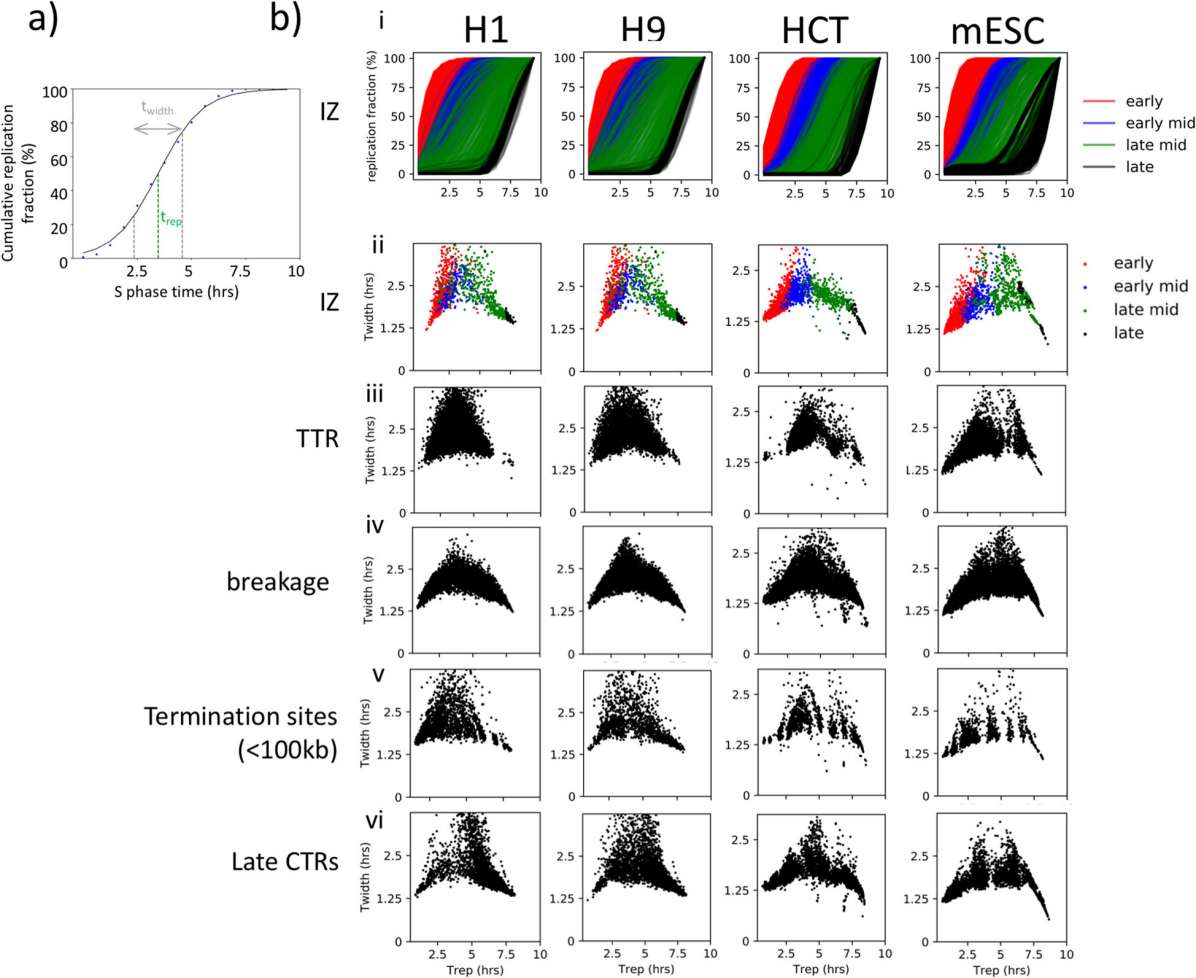
RT heterogeneity fluctuates throughout S phase and peaks in the middle of S phase. **a** Schematic showing the fitting of a sigmoidal curve on the bin wise cumulative percentage replicated plotted against time into S phase. Example datapoints (blue) of cumulative percentage of replication in high-resolution Repli-Seq heatmap. Black line represents the fitted sigmoidal curve. Green dotted line represents t_rep_ , which is the S phase time at which replication is 50% completed in the cell population. Distance between grey dotted lines represents t_width_, which is the time it takes for replication to be from 25% replicated to 75% replicated in the cell population. **b** (i) Cumulative replication percentage for IZs of early (red), early-mid (blue), late-mid (green) and late (black) timing where each line represents an individual IZ. **b** (ii) Scatter plots showing the relationship between *T*_width_ and *T*_rep_ calculated from fitted sigmoidal curves for early (red), early-mid (blue), late-mid (green) and late (black) IZs in H1, H9, HCT116 and F121-9 mESC. **b** (iii–vi) Scatter plots showing the relationship between *T*_width_ and *T*_rep_ for TTRs (iii), breakages (iv), termination sites (< 100 kb) (v) and late CTRs (vi) in H1, H9, HCT116 and F121-9 mESC

The strong enrichment of nascent DNA within only a few temporal windows of S phase indicates a high degree of cell-to-cell conservation for all kinetic features of the RT programme genome-wide. However, there are also regions where nascent DNA signal was spread over a broader temporal interval. To measure the degree of temporal variability across the genome and within the different patterns identified in Fig. 1d, we examined the relationship between two important indices of replication dynamics, *T*_rep_ (the time point at which 50% of all cells have finished replicating the locus) and *T*_width_ (the time difference between the locus being replicated in 25% of all cells and 75% of all cells) [34]. Since the 16 fraction heatmap values are representative of the percentage of cells in the population that have replicated the genomic bin in each time interval, we can estimate *T*_rep_ and *T*_width_ by performing a sigmoidal fit on the column-wise cumulative sum of the bin values (Fig. 3a). Briefly, the cumulative sum for any genomic locus is calculated as such: at S phase fraction 1 (S1), the cumulative sum equals the value the genomic bin assumes in S1, increases to the sum of S1 and S2 at the end of S2 and eventually reaches 100%, the sum of S1, S2, S3 through to S16 at the end of S phase. The value of *T*_width_ represents the variation in replication time. IZs were divided into four categories (early, early-mid, late-mid, late) as in Fig. 2a. For all cell types assayed, the *T*_width_ increased as S phase progressed towards mid S phase and decreased from mid S phase to late S phase (Fig. 3b (i, ii)). The relationship between *T*_width_ and *T*_rep_ of TTRs assumes a similar pattern to that of IZs. Variability is the highest for mid S phase for all features measured. Assuming a 10-h S phase, by converting 16 S phase fractions into fractions of 10 h, we found that almost all regions went from being 25% replicated to 75% replicated in 1.25–2.5 h. We conclude that the time at which each IZ will initiate replication is remarkably uniform within the majority of cells in the population. In fact, all features of the RT programme, including TTRs and termination sites (< 100 kb), which both appear devoid of origin activity by the measurements described above, also display the pattern of increased variability during mid S phase, consistent with their control by nearby IZs (Fig. 3b (iii, iv, v, vi)).

### IZs that initiate at different times have different chromatin features

**Fig. 4:**
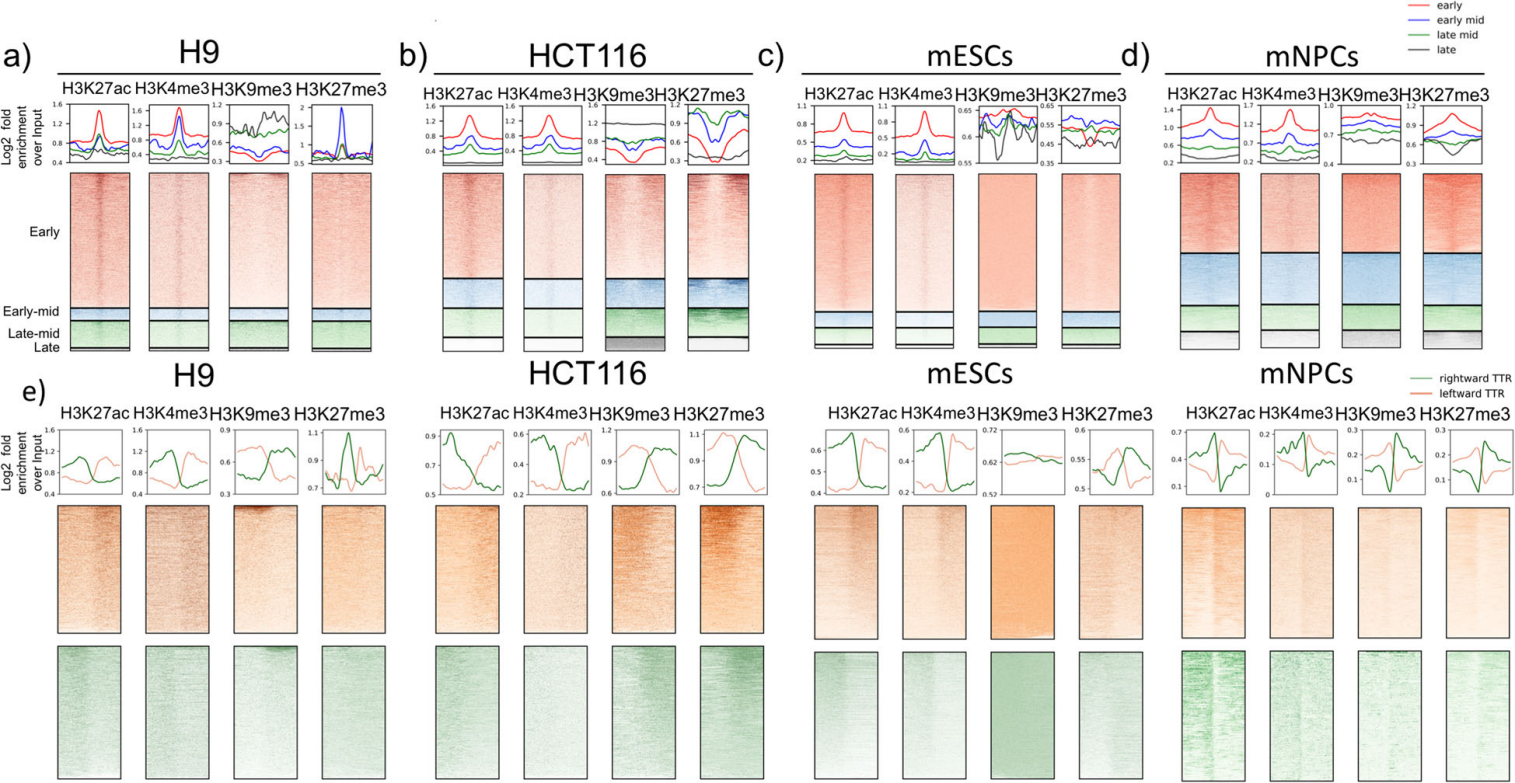
Correlation between IZs and histone modifications is cell type and timing specific. **a**–**d** Mean line plots (top row) and heatmaps (bottom row) of H3K27ac, H3K4me3, H3K9me3 and H3K27me3 fold enrichment signal centred on early (red), early-mid (blue), late-mid (green) and late (black) IZs ± 500 kb in H9 (**a**), HCT116 (**b**), mESC (**c**) and mNPC (**d**). **e** Mean line plots (top row) and heatmaps (bottom row) of H3K27ac, H3K4me3, H3K9me3 and H3K27me3 fold enrichment signal centred on TTRs ± 500 kb in H9, HCT116, mESC and mNPC. Green and orange lines/heatmaps indicate rightward and leftward TTRs, respectively

There have been numerous reports linking origin firing with active histone marks and active gene transcription [35]. Centring on IZs, we performed a series of pile-ups showing the enrichment of histone modifications H3K4me3, H3K27ac, H3K27me3 and H3K9me3 at IZs (Fig. 4a–d). Early, early-mid and late-mid IZs are enriched for H3K27ac, which marks active transcription start sites (TSSs) and enhancers, as well as H3K4me3, which marks TSSs. Levels of these active histone marks in the regions surrounding IZs were positively correlated with earlier replication timing. This correlation was consistent across all cell types, suggesting that these active marks are not necessary for initiation per se but correlate with early replication, possibly due to their correlation with other features such as transcription. By contrast, repressive histone marks are much more variable between cell types with respect to their enrichment at IZs or their correlation to replication timing, consistent with prior reports showing that differences in active but not repressive histone marks correlate with differences in replication timing between cell lines [36]. H3K9me3 shows a slight depletion in almost all IZs except for the late ones highlighting that there is no genome-wide correlation or anticorrelation between any of the queried histone marks and IZs. In H9 hESCs, consistent with its marking of bivalent promoters and concurrence with H3K4me3, H3K27me3 is enriched at early, early-mid and late-mid IZs (Fig. 4a). The highest concentration of H3K27me3 is found in late-mid IZs. In HCT116, however, H3K27me3 is depleted from early, early-mid and late-mid IZs with late IZs showing no depletion or enrichment (Fig. 4b). In mouse ESCs, H3K27me3 is depleted from only early IZs; it does not show depletion or enrichment for IZs of other timing (Fig. 4c). In mNPCs, however, H3K27me3 is mostly enriched in early IZs and depleted in late IZs (Fig. 4d). This variable relationship between IZs and histone modifications further highlights the complexity underlying the link between replication initiation and the local chromatin environment and the absence of a one-to-one correlation between initiation and any individual epigenetic mark.

TTRs, on the other hand, are depleted of active histone marks themselves and the surrounding histone modification patterns are consistent with the presence of IZs upstream and downstream of rightward and leftward TTRs, respectively (Fig. 4e). H3K4me3 and H3K27ac are enriched on the IZ side of the TTR for all cell lines. H3K9me3 and H3K27me3 show cell-type-specific features that echoes their relationship with IZs. For instance, mESC IZs show no depletion of H3K9me3 consistent with its even distribution across TTRs (Fig. 4c, e). HCT116, on the other hand, shows depletion of both H3K9me3 and H3K27me3 at its IZs, which is in agreement with its preferential colocalisation on the termination side of TTRs (Fig. 4b, e). Together, this shows that TTRs and IZs are differentiated by their distinct epigenetic features. TTRs lack the active marks that exist at IZs, which could potentially explain their lack of origin activity. Therefore, TTRs punctuate both chromatin marks and initiation potential.

Moreover, we show that sites of termination are associated with a paucity of active marks and enriched for repressive marks regardless of their sizes (Additional file 1: Fig. S12). Cell line-specific differences are also observed. For instance, H3K27me3 is enriched in both types of termination sites in HCT116 yet depleted in those in H1 and H9 hESCs. Overall, despite the origin activity present in late CTRs, they are enriched for repressive marks to the same extent as small termination sites (< 100 kb) that are devoid of origin activity suggesting that origins in late CTRs are activated independently from the enhancing effects of active marks. This is supported by the observation that late IZs (Fig. 4a–d) are the most similar of all IZs to the chromatin features of termination sites. Perhaps additional mechanisms come into play late in S phase to ensure completion of replication prior to mitosis.

#### Developmental regulation of replication timing by IZs

**Fig. 5:**
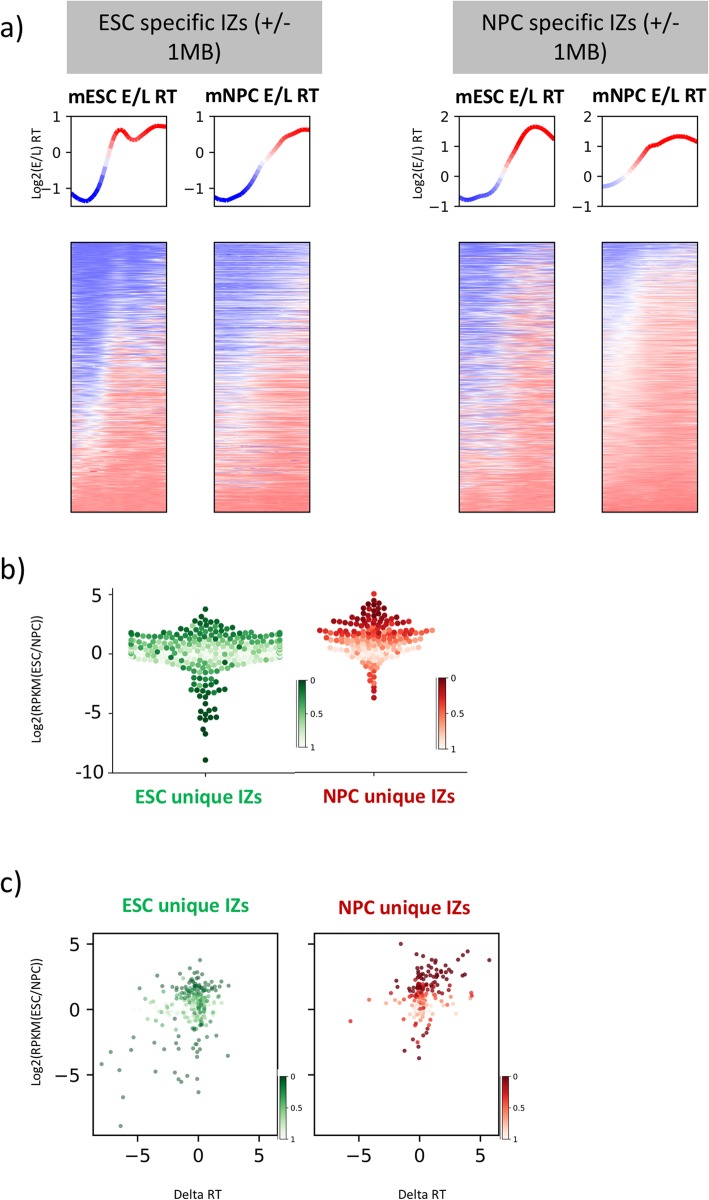
Developmentally controlled IZs show minimal RT switches and occasional transcriptional changes. **a** mESC and mNPC Log2E/L RT centred at ESC unique IZs (left panel) and NPC unique IZs (right panel) ± 1 MB. Line plots were colour-coded with blue and red representing negative and positive log2E/L RT. **b** Distributions of expression differences of genes overlapping with ESC and NPC unique IZs (calculated as log2(mESC RPKM/mNPC RPKM)). Expression differences were colour-coded by false discovery rate (FDR) from 0 to 1. **c** Scatter plots showing that the expression differences were colour-coded by FDR from 0 to 1

We next investigated whether high-resolution Repli-Seq could provide novel insight into the developmental control of RT. Changes in RT could be mediated by either turning IZs on or off or by changing the initiation time of specific IZs. We found that both mechanisms occur. First, we defined IZs that were active uniquely in mESCs or mNPCs (Fig. 2b). In the cell line where the IZs were not initiating, the loci were passively replicated by a nearby IZ and did not show significant log2 (E/L) RT change, remaining early replicating (Fig. 5a, Additional file 1: Fig. S13a). To determine whether cell-type-specific IZs are coordinately activated or suppressed in clusters, we calculated the median distance between mESC and mNPC unique IZs, which was approximately 2 MB, with the upper quartile being around 5 MB; therefore, in the majority of cases, developmental IZs were individually regulated (Additional file 1: Fig. S13b). Interestingly, loss of initiation activity was not always accompanied by the loss of transcriptional activity and vice versa (Fig. 5b) despite the general correlation between initiation/early replication with increased transcription [37, 38] (Fig. 5c). Overall, more NPC unique IZs were associated with increased transcription in mNPC than mESC unique IZs with increased transcription in mESC. Together, we show that developmentally regulated initiation activity is independent from transcription activation globally. To define the histone associations at developmentally controlled IZs, we examined H3K27ac localisation around mESC- and mNPC-specific IZs. We found that H3K27ac showed slightly higher enrichment in the cell line where the IZs initiated, albeit even in the cell line where the IZs did not initiate, H3K27ac was more enriched within the IZs than in the surrounding regions (Additional file 1: Fig. S13c). We subsequently identified IZs that were common between mESC and mNPC yet initiated at significantly different times (Additional file 1: Fig. S13d). H3K27ac showed similar trends of enrichment at these timing variable IZs as it did at cell-type-specific IZs (Additional file 1: Fig. S13e). Overall, we show that developmental IZs were controlled by regulation of both their initiation potential and timing, which was largely independent from transcriptional and epigenetic alterations on a genome-wide level.

### IZs form strong compartmental interactions

**Fig. 6:**
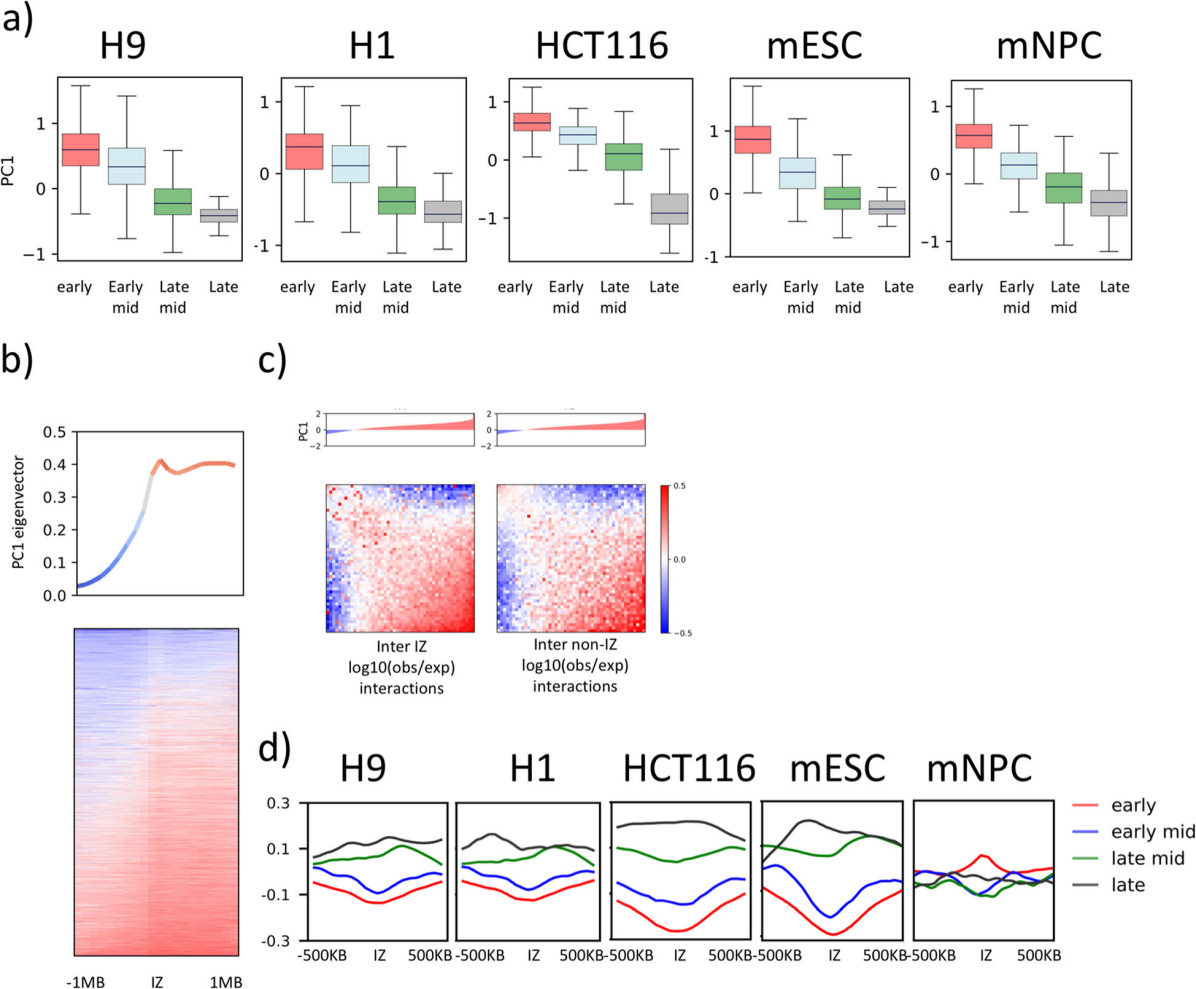
IZs form strong compartmental interactions. **a** Boxplot showing the distribution of PC1 values associated with IZs of early (red), early-mid (blue), late-mid (green) and late (black) timing in H9, H1, HCT116, mESC and mNPC. Black lines indicate medians of distribution. **b** Average PC1 eigenvector values (top) and pile-up heatmap of PC1 eigenvector values (bottom) centred on H1 IZs ± 1 MB. **c** Intra-IZ (left) and intra-control (bins in direct proximity to IZs occupying the same genomic space) (right) log10 (obs/exp) interactions binned according to the Hi-C PC1 values associated with the IZ from most the negative to the most positive. **d** Mean line plots of insulation scores centred at for IZs of early (red), early-mid (blue), late-mid (green) and late (black) timing ± 500 kb in H9, H1, HCT116, mESC and mNPC

A prior study has suggested the formation of long-range interaction hubs around replication origins [39]. We thus queried the A/B compartment in which IZs reside. Earliest IZs are mostly A compartment associating and those that initiate at later times in S phase are increasingly B compartment associating (Fig. 6a). However, IZs of all timing are more A compartment associating than their immediate surrounding regions (Fig. 6b), consistent with their preferential colocalisation with active histone marks compared to the neighbouring sequences (Fig. 4a). To investigate if IZs nucleate compartmental interactions, we compared IZ-IZ interactions that were binned and sorted into increasing PC1 intervals to interactions between non-IZ regions in immediate proximity to IZs that occupy the same genomic volume and show that IZs form stronger intra-compartmental (diagonal) interactions (Fig. 6c). At developmentally regulated IZs, the association with B compartment increased in the cell type where IZs became more late replicating showing that IZ timing changes were accompanied by concordant compartmental interaction changes (Additional file 1: Fig. S13f).

Active chromatin marks and active gene transcription have been shown to correlate with high insulation on Hi-C chromatin contact heatmaps, sometimes referred to as topologically associated domain (TAD) boundaries [40, 41]. Consistently, we find that early and early-mid IZs, which are characterised by active chromatin marks, also generally correspond to loci of high levels of insulation (Fig. 6d) while late IZs, which lack active histone marks, do not colocalise with increased insulation. Moreover, initiation zones *called* by [20] using OK-seq data were shown to fire preferentially at TAD boundaries and those were predominantly early replicating. Surprisingly, mNPCs did not conform to the correlation seen in hESCs, mESCs and HCT116 in that early IZs in mNPCs showed decreased insulation compared to IZs of later initiation timing (Fig. 6d). In summary, the correlation of IZs to TAD boundaries is incomplete and cell type specific, potentially driven indirectly by other factors that colocalise with IZs and share the same histone marks. It will be important to examine these correlations in other cell types.

### Biphasically replicated regions contain long transcribed genes, suggesting a link to genome fragility

**Fig. 7:**
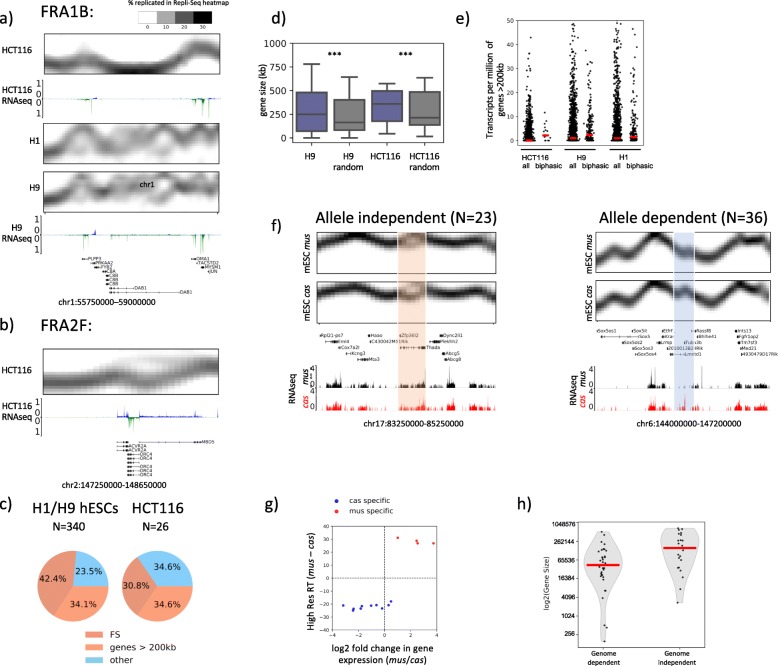
Biphasically replicated loci overlap with long genes that suggest genome fragility. **a** High-resolution Repli-Seq heatmaps and RNA-seq profiles for FRA1B (chr1:55,750,000–59,000,000), in HCT116, H1 and H9 hESCs. **b** High-resolution Repli-Seq heatmaps and RNA-seq profiles for FRA2F (chr2:147,250,000–148,650,000), a mapped HCT116 CFS, which is replicated biphasically in HCT116. **c** Fractions of biphasic loci overlapping with annotated CFS and long genes (> 200 kb) in hESCs and HCT116. **d** Boxplot showing distributions of gene sizes of those that overlap with biphasic sites in H9 and HCT116 (purple boxes) and distributions of gene sizes of those that overlap with randomly selected shuffled genome segments that occupied the same genomic space as biphasic sites (grey boxes) (Mann-Whitney *U* test ***p < 0.05). Distribution of TPM of genes > 200 kb genome-wide or genes > 200 kb overlapping with biphasic regions in HCT116, H9 and H1 hESCs. Red lines represent the medians of TPM. **f** F121-9 mESC allele resolved Repli-Seq heatmaps and RNA-seq tracks at genome independent (chr17:83,250,000-85,250,000) and dependent loci (chr6:144,000,000-147,200,000). Orange highlight indicates the genome-independent biphasic site in chr17, which actively transcribed on both *mus* and *cas* alleles. Blue highlight indicates the genome-dependent biphasic site in chr6, which is replicated earlier in *cas* allele accompanied by active transcription. **g** Correlation between log2 (gene expression differences) and RT differences of *mus* over *cas* calculated using high-resolution Repli-Seq according to the formula *mus* log 2(Sum(S1 ∗ 8, S2 ∗ 7, S3 ∗ 6, …S8 ∗ 1)/Sum(S9 ∗ 1, S10 ∗ 2, S11 ∗ 3…S16 ∗ 8)) − *cas* log 2(Sum(S1 ∗ 8, S2 ∗ 7, S3 ∗ 6, …S8 ∗ 1)/Sum(S9 ∗ 1, S10 ∗ 2, S11 ∗ 3…S16 ∗ 8)) at genome-dependent biphasic sites. **h** Distribution of log2(gene size) of genes overlapping with genome-dependent or genome-independent biphasic sites. Red lines represent the medians of log2(gene size)

We observed biphasic patterns of replication in H1, H9 hESCs and HCT116 (Fig. 7a, b), which are defined as regions with two distinct times of replication. We first considered whether these were regions of imprinting, known to show allele-specific asynchrony [42]. We found that some imprinted genes show smearing in Repli-Seq heatmaps, indicative of small and stochastic differences in the RT (Additional file 1: Fig. S14a). However, their RT differences did not reach our threshold of biphasic patterns, which required an intervening temporal interval when we could not detect replication of either allele. As a whole, the correlation between alleles at imprinted genes remained high and the majority of biphasic loci did not contain imprinted genes (Additional file 1: Fig. S14b). Surprisingly, however, we found that the biphasic sites in human cells were associated with common fragile sites (CFS) and/or transcribed large genes (> 200 kb), which are known to be correlated with genome instability and CFSs [43, 44]. This is also consistent with the prior observation [9] of one fragile site (FRA3B) that appeared to replicate biphasically in 6-stage Repli-seq. The biphasic locus shown in Fig. 7a does not show detectable biphasic replication in H1 hESC datasets from [9] (Additional file 1: Fig. S15a) showing that the temporal resolution achieved by our method is necessary for the comprehensive detection of replication bimodality. This is further supported by the comparison between Repli-Seq profiles of FRA3B from H1 hESC datasets from [9] and cell lines in this study shown in Additional file 1: Fig. S15b. Together, our results demonstrate that the temporal resolution afforded by datasets used in this study was necessary for the comprehensive identification of biphasic patterns genome-wide.

Biphasic replication sites were cell-type-specific, as were CFSs [43, 45]. We identified 340 biphasic replication sites in H1/H9 hESCs, 144 of which (42%) were CFSs (annotated in other cell lines) and 116 have not been shown to be fragile in any cell line but contained large genes (> 200 kb). HCT116 contained 26 biphasic sites. CFSs have been mapped cytogenetically in HCT116 [46], and 9 out of 10 of those that map to autosomes displayed biphasic replication patterns, validating the fragility of sites detected by high-resolution Repli-Seq (Fig. 7b, c). The single undetected CFS in HCT116 was fragile in only 1.3% of total CFS breaks. Ten of the remaining 17 HCT1216 biphasic sites that did not overlap with mapped CFSs nonetheless contained large genes or overlapped with CFSs mapped in other cell types and could be potential novel fragile sites. The overlap between biphasic sites and large genes was statistically significant (Fig. 7d). We found that biphasic regions were marked by active histone marks such as H3K4me3 and H3K27ac, which suggests that they were potentiated for transcription or under active transcription (Additional file 1: Fig. S15c). We compared the levels of transcription of large genes overlapping with biphasic loci against those of large genes genome-wide in HCT116, H9 and H1 hESCs and found the medians of transcripts per million (TPM) distribution were higher in biphasic genes, suggesting that they were generally expressed (Fig. 7e). In conclusion, biphasic patterns are a potential replication signature of genome fragility induced by transcribed long genes.

To determine whether bimodality arose from replication asynchrony between maternal and paternal alleles or replication asynchrony on a population level, we plotted separate maternal and paternal Repli-Seq heatmaps in hESC line H1 in which SNPs have been phased for the region [47] (Additional file 1: Fig. S16). The high similarity between maternal and paternal heatmaps for the locus shown in Fig. 7a and the biphasic patterns that were vaguely visible in allele-parsed heatmaps did not support an allelic difference argument, but the sparsity of SNPs in human cells posed a challenge for a statistically sound conclusion. For this reason, we examined the F121-9 cell line, which allows effective allele parsing due to its high SNP density. There are 59 sites in F121-9 mESCs that were found to be biphasically replicated, 36 of these were found to be due to allelic differences. The remaining 23 sites were found to be allele independent; the biphasic patterns were retained in heatmaps for individual alleles (Fig. 7f). Moreover, allele-dependent bimodality was associated with differential gene expressions between alleles whereas allele-independent biphasic sites exhibited equal transcription output from both alleles. In the example shown in Fig. 7f, the earlier replicating locus on the *cas* allele coincides with higher transcription of the Tuba3b gene on the same allele. The same trend holds true genome-wide in that the earlier replicating locus on either allele was associated with higher levels of gene transcription within the locus (Fig. 7g). In contrast to allele-independent loci, allele-dependent loci showed no preferential affiliation with large genes. Indeed, we find that larger genes correlate better with allele-independent than allele dependent biphasic sites (Fig. 7h). We conclude that biphasic replication at allele-dependent and allele-independent loci are orchestrated by distinct mechanisms, with allele-independent asynchrony associated with large expressed genes that in humans are linked to CFSs and allele-dependent asynchrony potentially encoded by polymorphic differences between the genomes.

## Discussion

The apparent paradox between highly stochastic replication origin usage in mammalian cells and the considerably more deterministic nature of their RT patterns has been the focus of many studies [29]. By using a short nascent DNA labelling time and increased number of S phase fractions, we have produced Repli-Seq profiles with exquisitely high temporal resolution, revealing finely choreographed temporal structure in genome replication that is remarkably uniform between cells in a population. The data reveal discrete initiation zones at 50-kb resolution that initiate at various time throughout S phase. Bi-directional replication forks can be detected emanating from all temporally distinct IZs but those with different initiation times are differentiated by characteristics such as SNS origins and chromatin mark enrichment. We show that when sparse regions of initiation, which escaped detection in previous datasets, are filtered out of TTRs, the remaining ‘true TTRs’ show tight distributions of gradients and can be accounted for by single, very long, uni-directional replication forks. When all features of the genome consistent with initiation are compiled, we estimate that > 70% of 50-kb segments of the genome harbour initiation sites. IZ activity and timing are both regulated during differentiation at the level of individual IZs. Finally, our data reveal previously undetected regions of replication bimodality some of which are linked to allelic polymorphisms and some that are sites of random asynchrony, the latter of which overlap with CFSs or large genes (> 200 kb) associated with genome instability.

We identified IZs as peaks in high-resolution Repli-Seq heatmaps throughout S phase, which are subsequently cross referenced with origin mapping methods such as SNS-seq and OK-seq. Early IZs show enrichment in SNS-seq while late IZs do not. On the contrary, IZs of all timing show the corresponding directionality switch in fork polarity in OK-seq data suggesting that high-resolution Repli-Seq and OK-seq identify initiation events of similar characteristics. Both methods can detect broad zones of initiation containing origin clusters as well as zones with one or a few more efficient sites that are detected at higher resolution by the SNS-seq method and so OK-seq and Repli-seq are less sensitive than SNS-seq to the decrease in individual origin efficiency as S phase progresses. In addition to providing cross-validation, Repli-Seq and OK-seq were complementary methods that could identify sets of IZs that eluded detection by the other method. Repli-Seq identified low efficiency IZs that manifested as shallow ascension in OK-seq RFD, thus were below the threshold of OK-seq IZ calling whereas OK-seq could identify IZs embedded in late CTRs that did not manifest as peaks in Repli-seq heatmaps. The enrichment of SNS-seq signal in early IZs is also consistent with the preferential colocalisation of early IZs with active histone marks, a defining characteristic of origins identified through SNS-seq. In accordance with the depletion of SNS-seq signal in late IZs, late IZs are also devoid of active histone marks. We also show cell-type-specific correlation between histone modifications and origin firing; thus, while a permissive chromatin environment might enhance the probability of firing and the formation of origin clusters manifesting as IZs, origins can fire in an environment defined by repressive histone marks.

In addition to IZs, we defined TTRs in high-resolution Repli-Seq heatmaps and approximated the fork speed that would give rise to the slopes seen in TTRs in a 10-h long S phase. The approximated speeds are in good concordance with the range of fork speeds obtained through DNA combing and other methods [27, 48]. The primary mode of replication in TTRs has been controversial, with some reports consistent with passive uni-directional fork movement [2] and others claiming that slopes of TTRs are too fast and must contain sequentially activated origins [14]. Our results demonstrate that prior datasets were not able to resolve true TTRs from small inefficient zones or shoulders of origin activity (breakages). High-resolution Repli-Seq allowed us to remove these breakages in the slopes, thus filtering for only true TTRs with continuous slopes. Overall, the human and mouse stem cell lines show very similar median TTR speed that is in the range of previously measured singular fork velocity and tight distribution of limited gradient variation in these cell types.

We estimated the relationship between *T*_width_ and *T*_rep_ by performing sigmoidal fitting on the 5 features identified from high-resolution Repli-Seq and found that mid S phase had the highest *T*_width,_ with early and late S phases showing decreased *T*_width_ representing decreased heterogeneity. This conclusion relies on the assumption that all bins finish replicating by the end of S16 and thus the sigmoidal curves finish at 100 for all bins including very late replicating regions. However, we cannot rule out the possibility that some cells did not finish replicating the very late regions, in which case the sigmoidal curves would have a larger *T*_width_. However, this observation is consistent with the correlation heatmap of high-resolution Repli-Seq showing lower correlation thus, more variations between middle S fractions (Additional file 1: Fig. S6b) as well as prior *T*_width_ calculations resulted from single-cell Repli-Seq using mid S phase cells [17]. Previous work [49] has shown increased initiation events in the middle S phase on a population level, which could potentially be the source of increased variation in terms of initiation time.

High-resolution Repli-Seq heatmaps reveal biphasic replication patterns that were previously undetected in E/L Repli-Seq. Surprisingly, these were not imprinted regions; imprinted regions showed very small and heterogeneous differences in RT. Biphasic sites can be either allele dependent or allele independent. Allele-dependent bimodality is typically associated with a higher gene transcription rate in the earlier initiating allele. However, not all transcriptional differences result in replication asynchrony and vice versa hence ruling out an absolute correlation between active transcription and replication initiation [17]. Allele-independent bimodality is enriched for large genes, which were implicated in genome fragility [50] and cytogenetically mapped CFSs. Previous studies have concluded that CFSs exhibit delayed replication by using E/L Repli-Seq [51]. However, E/L Repli-Seq produces an averaged RT profile, and a read enrichment in both early and late S phase fractions would render a locus seemingly mid-replicating. Through high-resolution Repli-Seq, we have found biphasic replication signatures, which could exhibit early peak bias, late peak bias or equal signal from both early and late peaks. Such signatures were associated with large genes and potential genome fragility and hence provide a means to predict novel CFSs. The precise cause for biphasic replication at large genes is elusive. Prior works have linked genome fragility with transcription-dependent replication delay at large genes [44, 50]. We hypothesise that the biphasic patterns are due to heterogeneous random mono-allelic levels of transcription at these large genes in the cell population causing varying extents of replication delay.

## Materials and methods

### Cell culture

H1 and H9 cells were cultured in mTeSR1 (StemCell Technologies #85850) on hESC-qualified Matrigel (Corning #354277)-coated dishes according to WiCell instruction. For the maintenance and expansion, cells were detached using ReLeSR (StemCell Technologies #95872). When the cell reached approximately 70% confluent, cells were pulse-labelled with 400 μM BrdU for 30 min. To obtain single-cell suspension easily, the BrdU-labelled H1 and H9 cells were harvested using Gentle Cell Dissociating Reagent (StemCell Technologies #07174). HCT116 cells were cultured in McCoy’s 5A medium supplemented with 10% FBS. When the cells were approximately 70% confluent, cells were pulse-labelled with 400 μM BrdU for 30 min. The BrdU-labelled HCT116 cells were harvested using trypsin-EDTA. F121-9 mouse embryonic stem cell was cultured in 2i media on gelatin-coated dish as described in [52]. F121-9 differentiation to NPC was performed using RHB-A (TakaraBio #Y40001) as described in [52] for 10 days. F121-9 ESC and NPC were pulse-labelled with 400 μM BrdU for 30 min and harvested using ESGRO Complete Accutase (Millipore Sigma #SF006). NPC differentiation was confirmed per 4DN standard by cell morphology as well as qPCR for marker genes: Oct4 (ESC marker), Dppa2/4 (ESC marker), Nestin (NPC marker) and Sox1 (NPC marker). For qPCR, total RNA was extracted using Direct-zol RNA Mini Prep (Zymo R2050) without DNase treatment. From 0.5 μg total RNA, cDNA was synthesised by ProtoScript® II First Strand cDNA Synthesis Kit (NEB E6560) using dT23 primer. Ct was normalised against GAPDH.

### E/L Repli-seq library preparation and sequence processing

The experiments and analyses of E/L Repli-Seq were carried out as described in [8]. Briefly, the libraries were sequenced on Hi-Seq 2500. The fastq reads were mapped to human genome hg38 or mouse genome mm10 with the parameters --no-mixed, --no-discordant. PCR duplicates were removed using samtools rmdup. Log2 E/L ratio was calculated for 50-kb bins. The final profiles were Loess smoothed and quantile normalised using all profiles used in this paper.

### 16 fraction high-resolution Repli-seq processing and library preparation

The BrdU-labelled cells were fixed in 70% ethanol and stained with propidium iodide as described in [8] then sorted by BD FACSAria SORP according to the DNA content. Eighty thousand cells were collected for each fraction. In order to obtain reproducible sorting windows easily, the region from G1 peak to mid-point between G2 peak and the end of G2 was equally sliced into 16 to make S1–S16 fractions (H1 and H9). As we found S15–S16 fractions did not contain detectable BrdU-labelled DNA, in the later experiments, the region from G1 peak to G2 peak was equally sliced into 16 to make S1–S16 fractions (HCT116, F121-9 ESC and NPC). Any cells on the left of G1 peak were considered non-replicating and collected as G1 fraction. From cells in each fraction, total genomic DNA was extracted. 0.1 ng of BrdU incorporated cross-species mitochondrial DNA (primers included in Additional file 2 Table S1) and 1 ng of BrdU negative cross-species DNA (primers included in Additional file 2 Table S1) were used as spike control and added to purified genomic DNA. qPCRs against spike-in DNA were performed to monitor BrdU pull-down efficiency. DNA from S fractions S1–S16 were made into libraries as described in [8] with the following modifications: after the genomic DNA was sheared and adaptors were ligated, adaptor-ligated DNA from 80K cells was used for BrdU immunoprecipitation (using 0.5 μg of anti-BrdU BD #555627 and 20 μg of anti-mouse IgG Sigma #M7023) and subsequently purified and indexed as previously described. For HCT116, F121-9 ESC and NPC, adaptor-ligated DNA from 80K cells was first incubated with 0.5 μg of anti-BrdU in 100 μL PSBT (0.137 M NaCl, 0.0027 M KCl, 0.01 M Na_2_HPO_4_, 0.0018 M KH_2_PO_4_, 0.1% Tween 20) for 20 min at room temperature, the BrdU-DNA/anti-BrdU complex was subsequently captured by 2 μL of Dynabeads Protein G (Thermofisher #10003D) directly added this reaction for 20 min at room temperature. The BrdU-DNA/anti-BrdU/Protein G bead complexes were washed with 200 μL of PBST for 3 times (5 min each) before the release of BrdU-DNA by Proteinase K digestion and purification described in [8] before library indexing.

### Sequencing, mapping and normalisation of high-resolution Repli-Seq data

Repli-Seq libraries were sequenced on Hi-Seq 2500. Reads were aligned to human genome hg38 or mouse mm10 using bowtie-2 with the same parameters as those used for E/L Repli-Seq. Reads per million (RPM) was calculated with 50-kb bin size for BrdU pull-down libraries of each S phase fraction as well as G1 control. The log2 ratio between RPM of BrdU pulldown and that of G1 WGS was calculated for each S phase fraction, which was subsequently used to construct a matrix consisting of 16 rows where each row represented an S phase fraction ranging from S1 to S16 and each column represented a 50-kb genomic bin. Fifty-kilobase genomic bin was chosen due to the following considerations: assuming a fork speed of 1.8 kb/min, 30 min of BrdU labelling would have enabled incorporation of the analogue in at least 50-kb DNA per fork. Therefore, we estimate the technical limit of resolution to be approx. 50 kb. Bins with values below zero, i.e. bins that were associated with less signal than the corresponding ones in G1 WGS, were converted to zero. Sex chromosomes were removed and excluded from further analyses. The Repli-Seq heatmap matrix was smoothed by applying a Gaussian filter with sigma of 1:

1 $$ \frac{1}{2\pi {\sigma}^2}{e}^{-\frac{x^2+{y}^2}{2{\sigma}^2}} $$

Sigma of 1 was chosen because it was the minimum kernel size so that over-smoothing could be avoided. Effectively, each genomic bin is smoothed using the values of its 8 neighbouring bins: 2 bins upstream and downstream of the target bin in the same S phase fraction, 3 bins in the previous S phase fraction and 3 bins in the ensuing S phase fraction. For the bins in the first and the last S phase fractions, the Repli-Seq heatmap maxis is padded column-wise with the first and last S phase fractions, respectively. Subsequently, assuming that all bins should finish replicating at the end of S phase and should therefore be given equal weight when summing column-wise across all 16 fractions, each column was assigned a total arbitrary value of 100 and Gaussian smoothed value for the bin was substituted by its original value divided by the column sum multiplied by 100.

### Hi-C analyses

Hi-C datasets are downloaded from sources as stated in Additional file 2 Table S2 and aligned to hg38 or mm10 using HICUP (https://www.bioinformatics.babraham.ac.uk/projects/hicup/). The raw bam files are converted to .cool files using cooler and raw contact matrices are normalised either using ICE available in cooler or distance normalised as log2 (observed/expected) contacts where the expected contacts are calculated as diagonal sum divided by the number of valid bins in the diagonal. Eigenvector decomposition is performed using the cooltool package (https://github.com/mirnylab/cooltools) and ranked using GC content. Insulation scores were calculated according to [53] as the sum of ICE normalised interactions within a diamond window size of 500 kb for every 5-kb bin.

### ChIP-seq analyses

ChIP-seq and Cut-and-Run reads were downloaded from GEO as stated in Additional file 2 Table S2 and aligned to hg38 or mm10 using bowtie2. The bam files from alignment were used as input for MACS2 to call peaks and generate fold enrichment bigwig files, which were used subsequently for heatmap generation in the context of feature alignment such as IZ or TTR alignment. These alignment heatmaps were generated by constructing a matrix with each row representing a feature centred on feature centres and each column representing a genomic bin. The values in the matrix represent the ChIP-seq fold enrichment signal at the defined genomic distance from the centre of the region of interest.

### SNS-seq and Okazaki fragment seq

The sources of SNS-seq raw reads are stated in Additional file 2 Table S2. SNS-seq was aligned to hg38 using bowtie-2 and further processed according to [54]. The source of OKseq signal bigwig files and called OKseq initiation zones for mESCs is stated in Additional file 2 Table S2. The files were converted to mm10 using UCSC utility liftOver.

### Defining 5 features in high-resolution Repli-Seq: IZs, TTRs, breakages, small termination sites (< 100 kb) and late CTRs

Features were identified through the clustering algorithm BIRCH proposed in [30] implemented in the python package scikit-learn (https://scikit-learn.org/stable/index.html). Briefly, the BIRCH algorithm is a memory effective clustering algorithm used for image classification. It constructs a clustering feature (CF) tree consisting of sub-CF trees where CF is defined as (*N*,LS,SS) where *N* is the number of items in the sub-clusters, LS being the linear sum of *N* points and SS is the square sum of *N* points. The algorithm returns a set of clusters that captures the main patterns of the dataset, each column in Repli-Seq heatmap (i.e. each genomic bin) is assigned to a cluster that is characterised by a cluster centroid as shown in Additional file 1: Fig. S8. Features were identified depending on the S phase fractions where the maxima of the cluster centroids were located relative to the neighbouring bins. IZs were identified as consecutive bins, which were assigned to the same cluster and flanked by upstream and downstream bins that were assigned to later clusters. Leftward TTRs were identified as consecutive bins that were assigned to increasingly earlier clusters and were flanked by upstream and downstream bins that were assigned to later and earlier clusters, respectively. Rightward TTRs were identified as consecutive bins that were assigned to increasingly later clusters and were flanked by upstream and downstream bins that were assigned to earlier and later clusters, respectively. Breakages in TTR were identified as consecutive bins flanked on both sides by TTRs and were assigned to the same cluster. Small termination sites (< 100 kb) were identified as 1 or 2 50-kb bins flanked by upstream and downstream bins that were assigned to earlier clusters. Late CTRs were identified as consecutive bins (> 2) which were assigned to the same cluster and flanked by upstream and downstream bins that were assigned to earlier clusters.

### Timing heterogeneity estimation

The degree of heterogeneity of replication timing of IZs and TTRs was estimated by performing a sigmoidal fitting on the column-wise cumulative replication percentage. Briefly, the sigmoidal function

2 $$ f\left(\mathrm{x}\right)=\frac{e^{- kx}}{1+{e}^{- kx}} $$

was fitted using curve_fit function in scipy, which minimises the mean squared error. *T*_rep_ used in the timing-variation measurement is *f*(*x*) when *x* is 0.5 which means the bin is 50% replicated in the cell population and *T*_width_ used in the timing-variation measurement is *f*(0.75) – *f*(0.25) which is the time difference between 75% replicated and 25% replicated for any genomic bin.

### Identification of biphasically replicating regions and definition of overlap with CFS and long genes

Biphasically replicating regions are characterised by the presence of two maxima in the column-wise cumulative sum for any bin in normalised Repli-Seq heatmaps. Therefore, continuous bins that had two maxima in their column-wise cumulative sum were merged and identified as biphasic regions. Overlap of biphasic sites with CFS or long genes was defined as at least half of continuous genomic bins of biphasic sites being contained within corresponding CFSs or long genes.

### RNA-seq analyses

RNA-seq data for HCT116 and H9 were downloaded from the sources stated in Additional file 2 Table S2 and aligned to hg38 using STAR to generate bigwig signal files and reads per gene counts. mESC RNA-seq was downloaded from 4DN data portal (Additional file 2 Table S2) and aligned to mm10 using STAR. Allele parsing was performed on parsed bam files using SNPsplit with the SNP VCF file downloaded from https://www.sanger.ac.uk/science/data/mouse-genomes-project. TPM is generated based on the output SAM files from STAR using RSEM (https://deweylab.github.io/RSEM/).

### CFS database

Genomic coordinates of human CFSs used to overlap with biphasically replicating loci were from https://webs.iiitd.edu.in/raghava/humcfs/. Cytogenetically mapped HCT116-specific fragile sites were obtained from [46].

## Supplementary information

**Additional file 1: Figure S1.** Validation of NPC differentiation by qPCR using primers against Oct4, Dppa2, Nestin, Sox1. **Figure S2.** Validation of BrdU pull-down by qPCR using primers against alpha- and beta globin. **Figure S3.** RPM of each S phase fraction is corrected using G1 WGS. **Figure S4.** G1 mappability control fraction was devoid of DNA replication. **Figure S5.** Normalisation of Repli-Seq heatmaps preserves signal. **Figure S6.** Percentage of replication in S1-S16 for top 10% earliest and latest replicated E/L Repli-Seq bins in mESC, mNPC, H1, H9 and HCT116. **Figure S7.** Comparison between H1 hESC datasets from [9] and H1 hESC datasets from this work. **Figure S8.** Correlation heatmaps showing concordance between High-Resolution Repli-Seq datasets of human and mouse cell lines. **Figure S9.** Schematic showing the identification of replication features using BIRCH. **Figure S10.** OK-seq IZs are primarily early replicating. **Figure S11.** SNS-seq signal centred around rightward (dark green) and rightward (orange) TTRs in HCT116, H9 and mESCs. **Figure S12.** Mean line plots of H3K27ac, H3K4me3, H3K9me3 and H3K27me3 fold enrichment signal centred on late CTRs and termination sites (< 100 kb) +/− 500 kb in HCT116, H1 and H9. **Figure S13.** Identification of developmentally regulated IZs in mESC and mNPC. **Figure S14** Imprinted genes do not exhibit biphasic patterns. **Figure S15** Biphasic sites overlap with CFSs and are enriched for active histone marks. **Figure S16** H1 hESC unparsed and allele-parsed Repli-Seq heatmaps for chr1:55,750,000 – 59,000,000, the locus shown in Fig. 6a.

**Additional file 2 : Table S1** Primer lists for human and mouse. **Table S2** Resource table stating sources of external datasets.

## Data Availability

Accession codes for all datasets used in the paper can be found in Additional file 2: Table S2. All data generated in this study have been deposited to the GEO depository (GSE137764) [55]. The python code for data processing can be found on https://github.com/oliviacamel/High-Resolution-RepliSeq [56] and is released under Apache License 2.0.

## Glossary

**Table:**

Abbreviation	Term	Explanation
CTR	Constant timing region	CTRs are synchronously replicating regions that span hundreds of kilobases to megabases.
IZ	Initiation zone	IZs are regions of DNA containing multiple spatially clustered sites of replication initiation.
RFD	Replication fork directionality	RFD is calculated as the difference between rightward and leftward replication forks as measured by Okazaki fragment sequencing.
TTR	Timing transition region	TTRs are slopes in Repli-Seq data connecting early and late CTRs.

## References

[1] Rhind N, Gilbert DM (2013). DNA replication timing. Cold Spring Harb Perspect Biol.

[2] Hiratani I, Ryba T, Itoh M, Yokochi T, Schwaiger M, Chang CW (2008). Global reorganization of replication domains during embryonic stem cell differentiation. PLoS Biol.

[3] Rivera-Mulia JC, Buckley Q, Sasaki T, Zimmerman J, Didier RA, Nazor K (2015). Dynamic changes in replication timing and gene expression during lineage specification of human pluripotent stem cells. Genome Res.

[4] Moindrot B, Audit B, Klous P, Baker A, Thermes C, de Laat W (2012). 3D chromatin conformation correlates with replication timing and is conserved in resting cells. Nucleic Acids Res.

[5] Sima J, Chakraborty A, Dileep V, Michalski M, Klein KN, Holcomb NP (2019). Identifying cis elements for spatiotemporal control of mammalian DNA replication. Cell..

[6] Pope BD, Ryba T, Dileep V, Yue F, Wu W, Denas O (2014). Topologically associating domains are stable units of replication-timing regulation. Nature..

[7] Muller CA, Nieduszynski CA (2017). DNA replication timing influences gene expression level. J Cell Biol.

[8] Marchal C, Sasaki T, Vera D, Wilson K, Sima J, Rivera-Mulia JC (2018). Genome-wide analysis of replication timing by next-generation sequencing with E/L Repli-seq. Nat Protoc.

[9] Hansen RS, Thomas S, Sandstrom R, Canfield TK, Thurman RE, Weaver M (2010). Sequencing newly replicated DNA reveals widespread plasticity in human replication timing. Proc Natl Acad Sci U S A.

[10] Chen CL, Rappailles A, Duquenne L, Huvet M, Guilbaud G, Farinelli L (2010). Impact of replication timing on non-CpG and CpG substitution rates in mammalian genomes. Genome Res.

[11] Desprat R, Thierry-Mieg D, Lailler N, Lajugie J, Schildkraut C, Thierry-Mieg J (2009). Predictable dynamic program of timing of DNA replication in human cells. Genome Res.

[12] Koren A, Handsaker RE, Kamitaki N, Karlic R, Ghosh S, Polak P (2014). Genetic variation in human DNA replication timing. Cell..

[13] Dileep V, Rivera-Mulia JC, Sima J, Gilbert DM (2015). Large-scale chromatin structure-function relationships during the cell cycle and development: insights from replication timing. Cold Spring Harb Symp Quant Biol.

[14] Guilbaud G, Rappailles A, Baker A, Chen CL, Arneodo A, Goldar A (2011). Evidence for sequential and increasing activation of replication origins along replication timing gradients in the human genome. PLoS Comput Biol.

[15] Farkash-Amar S, Lipson D, Polten A, Goren A, Helmstetter C, Yakhini Z (2008). Global organization of replication time zones of the mouse genome. Genome Res.

[16] Dileep V, Gilbert DM (2018). Single-cell replication profiling to measure stochastic variation in mammalian replication timing. Nat Commun.

[17] Takahashi S, Miura H, Shibata T, Nagao K, Okumura K, Ogata M (2019). Genome-wide stability of the DNA replication program in single mammalian cells. Nat Genet.

[18] Hyrien O (2015). Peaks cloaked in the mist: the landscape of mammalian replication origins. J Cell Biol.

[19] Gilbert DM (2001). Making sense of eukaryotic DNA replication origins. Science..

[20] Petryk N, Kahli M, d’Aubenton-Carafa Y, Jaszczyszyn Y, Shen Y, Silvain M (2016). Replication landscape of the human genome. Nat Commun.

[21] Petryk N, Dalby M, Wenger A, Stromme CB, Strandsby A, Andersson R (2018). MCM2 promotes symmetric inheritance of modified histones during DNA replication. Science..

[22] Fu H, Besnard E, Desprat R, Ryan M, Kahli M, Lemaitre JM (2014). Mapping replication origin sequences in eukaryotic chromosomes. Curr Protoc Cell Biol.

[23] Besnard E, Babled A, Lapasset L, Milhavet O, Parrinello H, Dantec C (2012). Unraveling cell type-specific and reprogrammable human replication origin signatures associated with G-quadruplex consensus motifs. Nat Struct Mol Biol.

[24] Cayrou C, Ballester B, Peiffer I, Fenouil R, Coulombe P, Andrau JC (2015). The chromatin environment shapes DNA replication origin organization and defines origin classes. Genome Res.

[25] Smith DJ, Whitehouse I (2012). Intrinsic coupling of lagging-strand synthesis to chromatin assembly. Nature..

[26] Demczuk A, Gauthier MG, Veras I, Kosiyatrakul S, Schildkraut CL, Busslinger M (2012). Regulation of DNA replication within the immunoglobulin heavy-chain locus during B cell commitment. PLoS Biol.

[27] Anglana M, Apiou F, Bensimon A, Debatisse M (2003). Dynamics of DNA replication in mammalian somatic cells: nucleotide pool modulates origin choice and interorigin spacing. Cell..

[28] Klein K, Wang W, Borrman T, Chan S, Zhang D, Weng Z, et al. Genome-wide identification of early-firing human replication origins by optical replication mapping. bioRxiv. 2017:214841.

[29] Rhind N, Yang SC, Bechhoefer J (2010). Reconciling stochastic origin firing with defined replication timing. Chromosom Res.

[30] Zhang T, Ramakrishnan R, Livny M (1996). BIRCH: an efficient data clustering method for very large databases. SIGMOD Rec.

[31] Conti C, Sacca B, Herrick J, Lalou C, Pommier Y, Bensimon A (2007). Replication fork velocities at adjacent replication origins are coordinately modified during DNA replication in human cells. Mol Biol Cell.

[32] Pereira PD, Serra-Caetano A, Cabrita M, Bekman E, Braga J, Rino J (2017). Quantification of cell cycle kinetics by EdU (5-ethynyl-2′-deoxyuridine)-coupled-fluorescence-intensity analysis. Oncotarget..

[33] Wilson KA, Elefanty AG, Stanley EG, Gilbert DM (2016). Spatio-temporal re-organization of replication foci accompanies replication domain consolidation during human pluripotent stem cell lineage specification. Cell Cycle.

[34] Yang SC, Rhind N, Bechhoefer J (2010). Modeling genome-wide replication kinetics reveals a mechanism for regulation of replication timing. Mol Syst Biol.

[35] Fragkos M, Ganier O, Coulombe P, Mechali M (2015). DNA replication origin activation in space and time. Nat Rev Mol Cell Biol.

[36] Yue F, Cheng Y, Breschi A, Vierstra J, Wu W, Ryba T (2014). A comparative encyclopedia of DNA elements in the mouse genome. Nature..

[37] Cayrou C, Coulombe P, Vigneron A, Stanojcic S, Ganier O, Peiffer I (2011). Genome-scale analysis of metazoan replication origins reveals their organization in specific but flexible sites defined by conserved features. Genome Res.

[38] Comoglio F, Schlumpf T, Schmid V, Rohs R, Beisel C, Paro R (2015). High-resolution profiling of Drosophila replication start sites reveals a DNA shape and chromatin signature of metazoan origins. Cell Rep.

[39] Boulos RE, Arneodo A, Jensen P, Audit B (2013). Revealing long-range interconnected hubs in human chromatin interaction data using graph theory. Phys Rev Lett.

[40] Bonev B, Mendelson Cohen N, Szabo Q, Fritsch L, Papadopoulos GL, Lubling Y (2017). Multiscale 3D genome rewiring during mouse neural development. Cell..

[41] Dixon JR, Selvaraj S, Yue F, Kim A, Li Y, Shen Y (2012). Topological domains in mammalian genomes identified by analysis of chromatin interactions. Nature..

[42] Donley N, Thayer MJ (2013). DNA replication timing, genome stability and cancer: late and/or delayed DNA replication timing is associated with increased genomic instability. Semin Cancer Biol.

[43] Debatisse M, Rosselli F (2019). A journey with common fragile sites: from S phase to telophase. Genes Chromosomes Cancer.

[44] Brison O, El-Hilali S, Azar D, Koundrioukoff S, Schmidt M, Nahse V (2019). Transcription-mediated organization of the replication initiation program across large genes sets common fragile sites genome-wide. Nat Commun.

[45] Letessier A, Millot GA, Koundrioukoff S, Lachages AM, Vogt N, Hansen RS (2011). Cell-type-specific replication initiation programs set fragility of the FRA3B fragile site. Nature..

[46] Le Tallec B, Millot GA, Blin ME, Brison O, Dutrillaux B, Debatisse M (2013). Common fragile site profiling in epithelial and erythroid cells reveals that most recurrent cancer deletions lie in fragile sites hosting large genes. Cell Rep.

[47] Selvaraj S, RD J, Bansal V, Ren B (2013). Whole-genome haplotype reconstruction using proximity-ligation and shotgun sequencing. Nat Biotechnol.

[48] Fu H, Martin MM, Regairaz M, Huang L, You Y, Lin CM (2015). The DNA repair endonuclease Mus81 facilitates fast DNA replication in the absence of exogenous damage. Nat Commun.

[49] Goldar A, Marsolier-Kergoat MC, Hyrien O (2009). Universal temporal profile of replication origin activation in eukaryotes. PLoS One.

[50] Blin M, Le Tallec B, Nahse V, Schmidt M, Brossas C, Millot GA (2019). Transcription-dependent regulation of replication dynamics modulates genome stability. Nat Struct Mol Biol.

[51] Pelliccia F, Bosco N, Curatolo A, Rocchi A (2008). Replication timing of two human common fragile sites: FRA1H and FRA2G. Cytogenet Genome Res.

[52] Rivera-Mulia JC, Dimond A, Vera D, Trevilla-Garcia C, Sasaki T, Zimmerman J (2018). Allele-specific control of replication timing and genome organization during development. Genome Res.

[53] Mizuguchi T, Fudenberg G, Mehta S, Belton JM, Taneja N, Folco HD (2014). Cohesin-dependent globules and heterochromatin shape 3D genome architecture in S. pombe. Nature..

[54] Smith OK, Kim R, Fu H, Martin MM, Lin CM, Utani K (2016). Distinct epigenetic features of differentiation-regulated replication origins. Epigenetics Chromatin.

[55] Zhao PA, Sasaki T, Gilbert DM. High resolution Repli-Seq defines the temporal choreography of initiation, elongation and termination of replication in mammalian cells. GSE137764. 2019. Gene Expression Omnibus. https://www.ncbi.nlm.nih.gov/geo/query/acc.cgi?acc=GSE137764. Accessed 9 Mar 2020. 10.1186/s13059-020-01983-8 PMC7092589 32209126

[56] Zhao PA, Sasaki T, Gilbert DM. High resolution Repli-Seq defines the temporal choreography of initiation, elongation and termination of replication in mammalian cells. 2020. Github. https://github.com/oliviacamel/High-Resolution-RepliSeq. Accessed 9 Mar 2020. 10.1186/s13059-020-01983-8 PMC7092589 32209126

