# Supplementary material for: High-resolution Repli-Seq defines the temporal choreography of initiation, elongation and termination of replication in mammalian cells
Source: Genome Biol. 2020 Mar 24;21:76. doi: 10.1186/s13059-020-01983-8 (PMC7092589; doi:10.1186/s13059-020-01983-8)
Supplement: Supplementary file 1 — Additional file 1: Figure S1. Validation of NPC differentiation by qPCR using primers against Oct4, Dppa2, Nestin, Sox1. Figure S2. Validation of BrdU pull-down by qPCR using primers against alpha- and beta globin. Figure S3. RPM of each S phase fraction is corrected using G1 WGS. Figure S4. G1 mappability control fraction was devoid of DNA replication. Figure S5. Normalisation of Repli-Seq heatmaps preserves signal. Figure S6. Percentage of replication in S1-S16 for top 10% earliest and latest replicated E/L Repli-Seq bins in mESC, mNPC, H1, H9 and HCT116. Figure S7. Comparison between H1 hESC datasets from [9] and H1 hESC datasets from this work. Figure S8. Correlation heatmaps showing concordance between High-Resolution Repli-Seq datasets of human and mouse cell lines. Figure S9. Schematic showing the identification of replication features using BIRCH. Figure S10. OK-seq IZs are primarily early replicating. Figure S11. SNS-seq signal centred around rightward (dark green) and rightward (orange) TTRs in HCT116, H9 and mESCs. Figure S12. Mean line plots of H3K27ac, H3K4me3, H3K9me3 and H3K27me3 fold enrichment signal centred on late CTRs and termination sites (< 100 kb) +/− 500 kb in HCT116, H1 and H9. Figure S13. Identification of developmentally regulated IZs in mESC and mNPC. Figure S14 Imprinted genes do not exhibit biphasic patterns. Figure S15 Biphasic sites overlap with CFSs and are enriched for active histone marks. Figure S16 H1 hESC unparsed and allele-parsed Repli-Seq heatmaps for chr1:55,750,000 – 59,000,000, the locus shown in Fig. 6a. [file 13059_2020_1983_MOESM1_ESM.pdf]

Fig S1

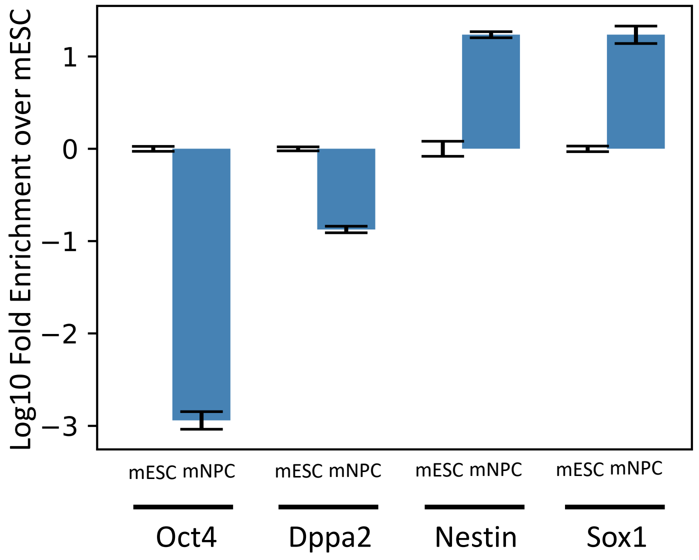

Log10 Fold Enrichment over mESC
1
0
−1
−2
−3
mESC mNPC
mESC mNPC
mESC mNPC
mESC mNPC
Oct4
Dppa2
Nestin
Sox1

Fig S2

a)

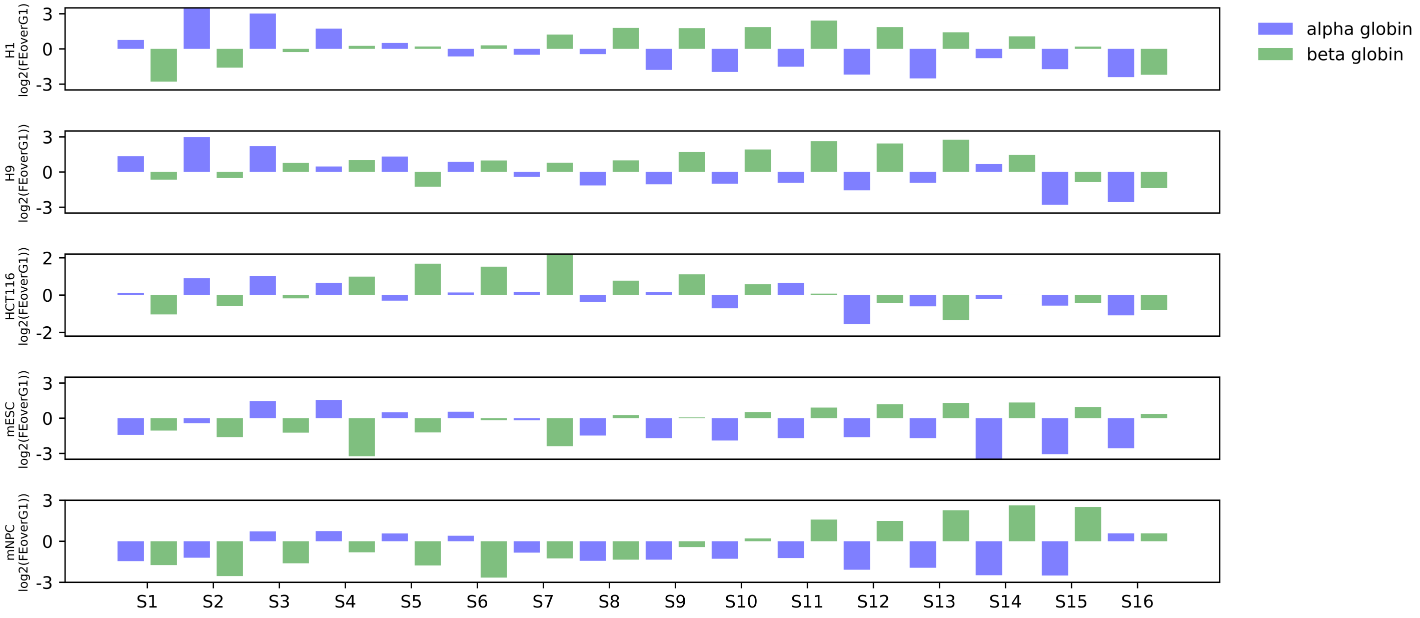
alpha globin
beta globin
H1 log2(FEoverG1)
H9 log2(FEoverG1))
HCT116 log2(FEoverG1))
mESC log2(FEoverG1))
mNPC log2(FEoverG1))
S1
S2
S3
S4
S5
S6
S7
S8
S9
S10
S11
S12
S13
S14
S15
S16

b)

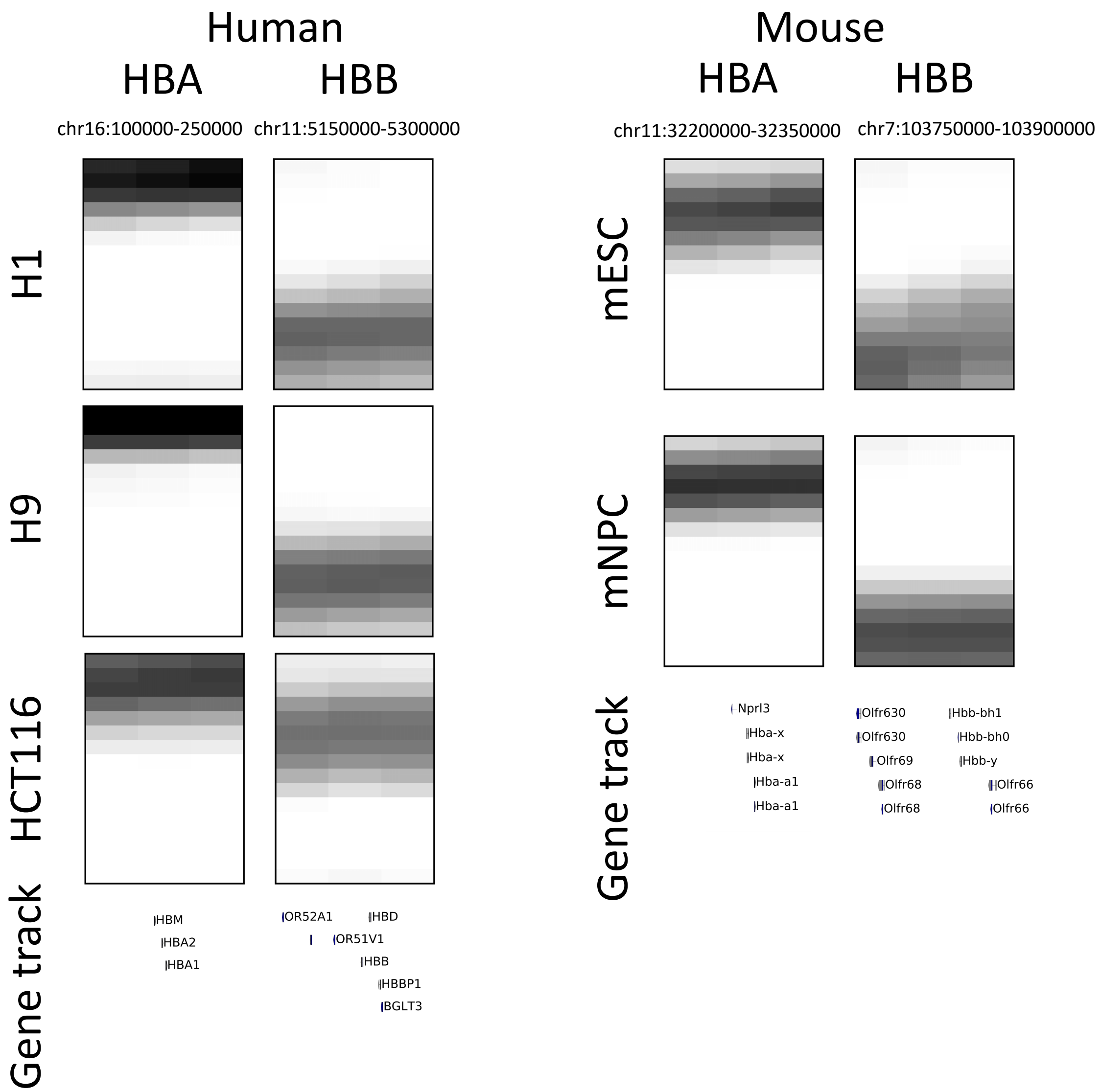
Human
Mouse
HBA
HBB
HBA
HBB
chr16:100000-250000
chr11:5150000-5300000
chr11:32200000-32350000
chr7:103750000-103900000
H1
H9
HCT116
Gene track
mESC
mNPC
Gene track
HBM
HBA2
HBA1
OR52A1
HBD
OR51V1
HBB
HBBP1
BGLT3
Nprl3
Hba-x
Hba-x
Hba-a1
Hba-a1
Olfr630
Olfr630
Olfr69
Olfr68
Olfr68
Hbb-bh1
Hbb-bh0
Hbb-y
Olfr66
Olfr66

Fig S3

a)

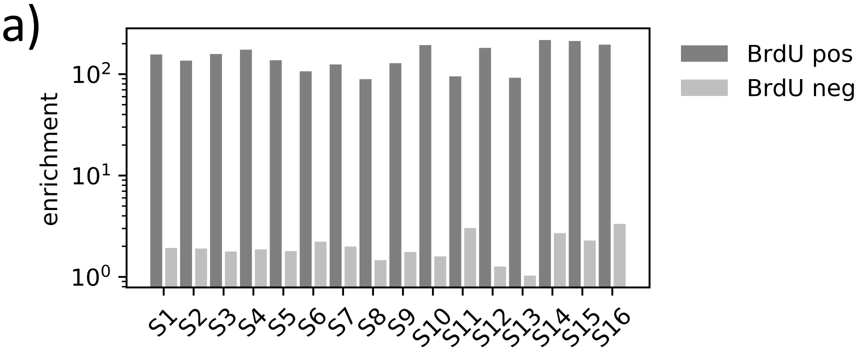
enrichment
BrdU pos
BrdU neg
S1 S2 S3 S4 S5 S6 S7 S8 S9 S10 S11 S12 S13 S14 S15 S16

b)

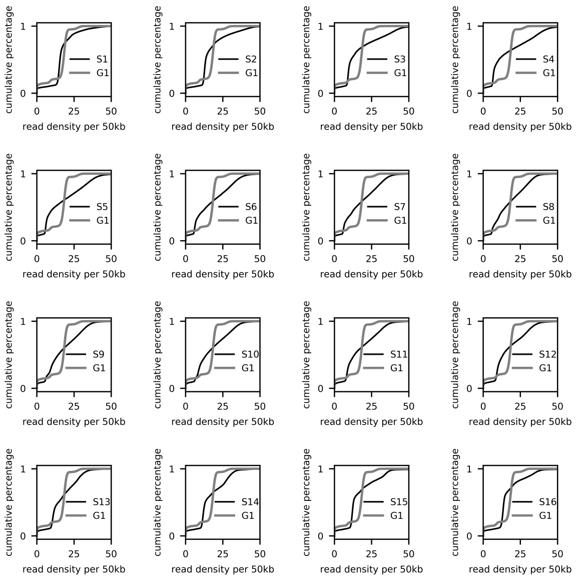
cumulative percentage
read density per 50kb
G1

c)

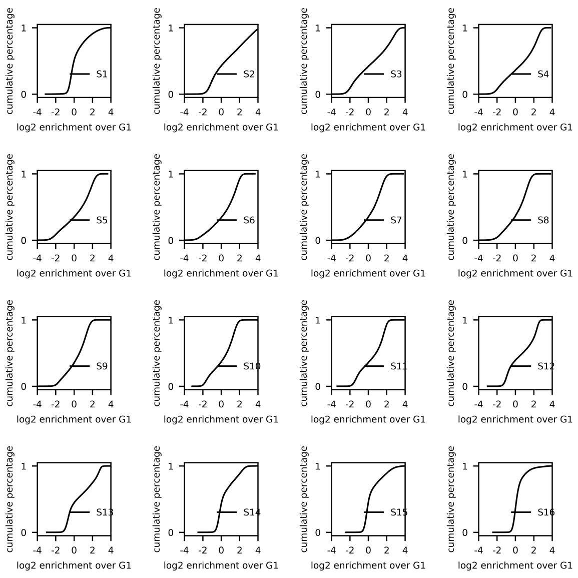
cumulative percentage
log2 enrichment over G1

d)

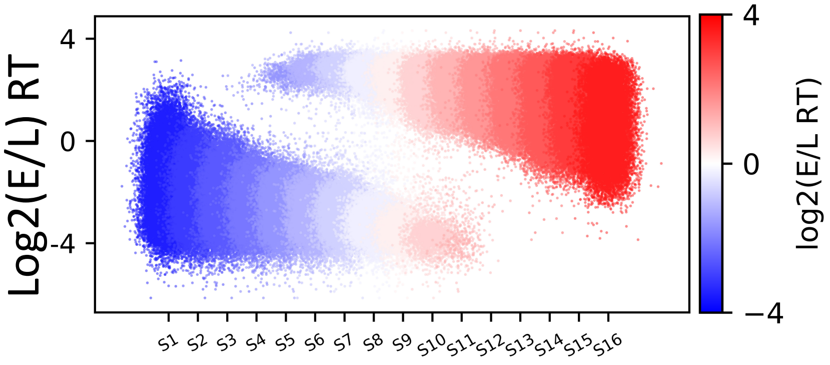
Log2(E/L) RT
log2(E/L RT)
S1 S2 S3 S4 S5 S6 S7 S8 S9 S10 S11 S12 S13 S14 S15 S16

Fig S4

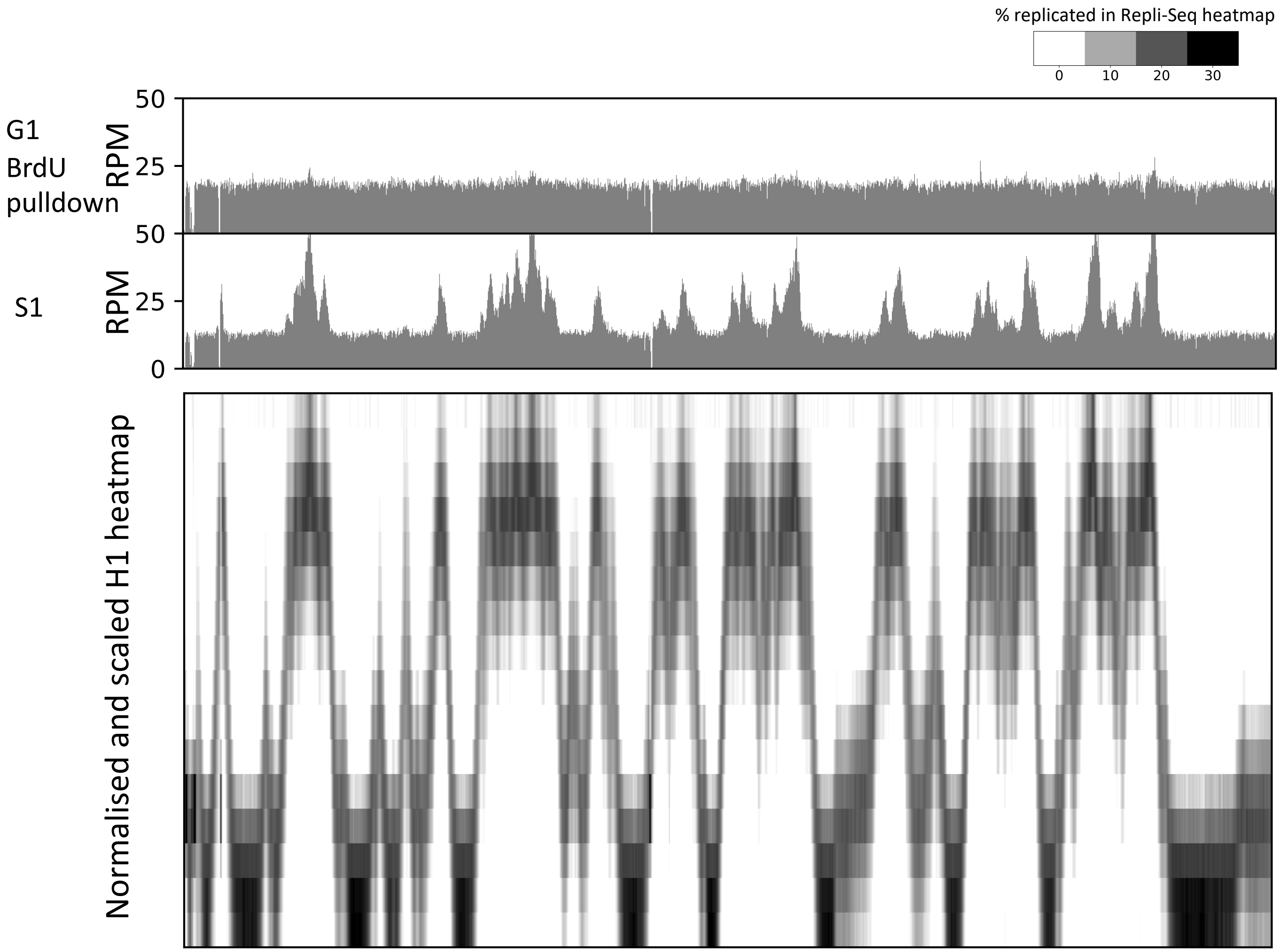

% replicated in Repli-Seq heatmap
0
10
20
30
G1
BrdU
pulldown
RPM
50
25
S1
RPM
50
25
0
Normalised and scaled H1 heatmap

Fig S5

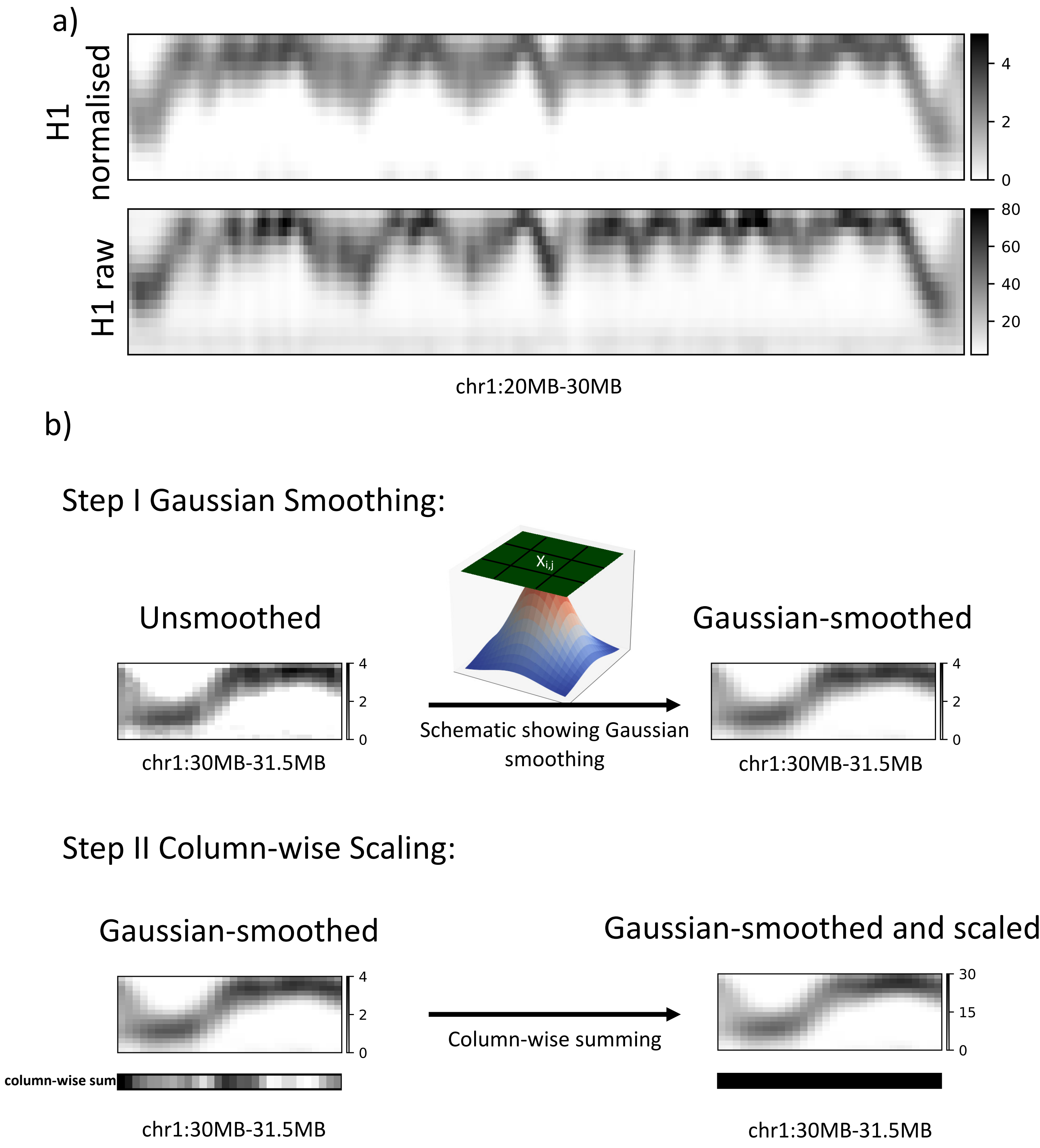
a)
H1 normalised
H1 raw
chr1:20MB-30MB
b)
Step I Gaussian Smoothing:
Unsmoothed
Gaussian-smoothed
chr1:30MB-31.5MB
Schematic showing Gaussian smoothing
Step II Column-wise Scaling:
Gaussian-smoothed
Gaussian-smoothed and scaled
Column-wise summing
column-wise sum
chr1:30MB-31.5MB

Fig S6

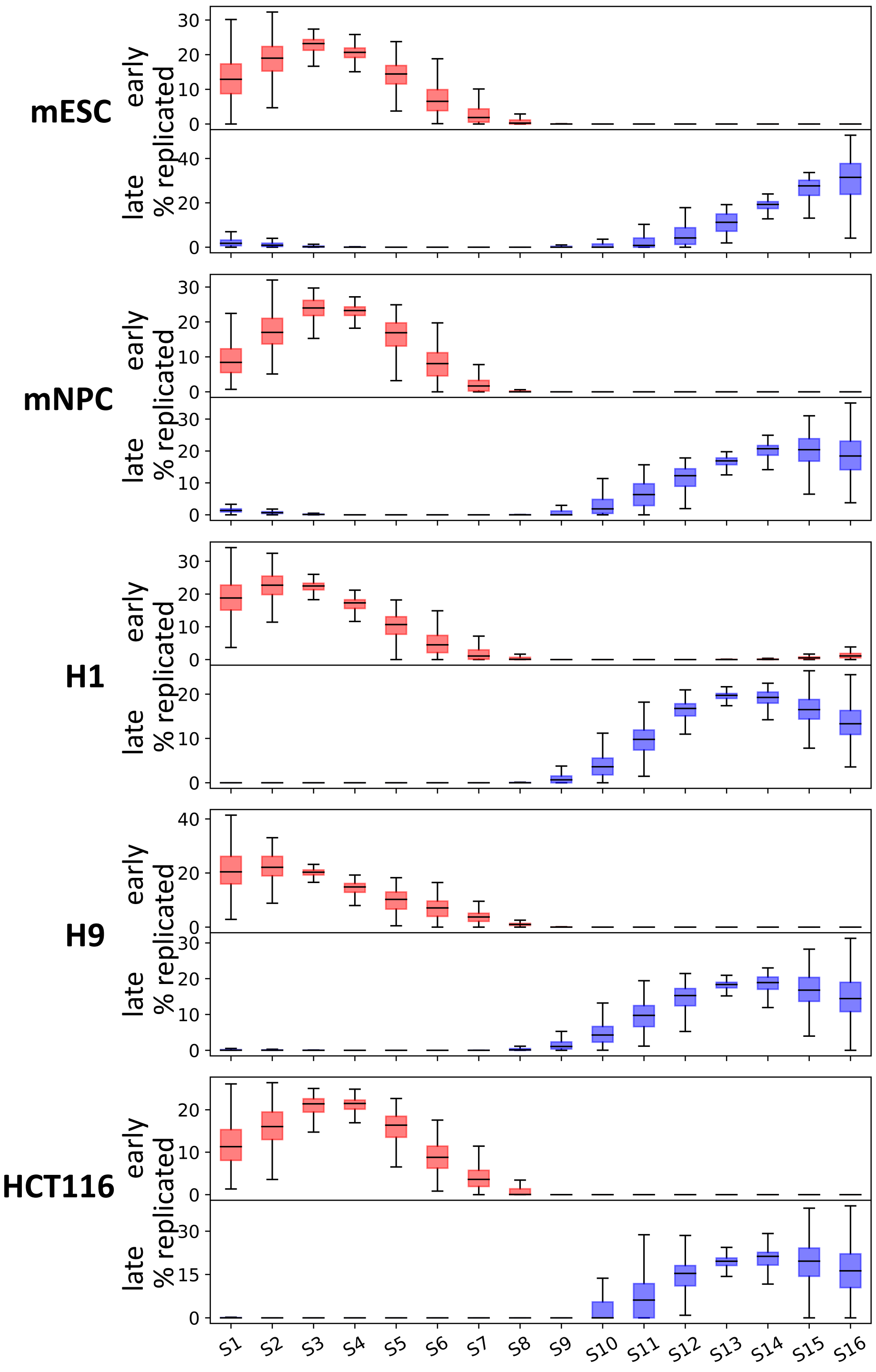
mESC
mNPC
H1
H9
HCT116
early
late
% replicated
S1
S2
S3
S4
S5
S6
S7
S8
S9
S10
S11
S12
S13
S14
S15
S16

Fig S7

a)

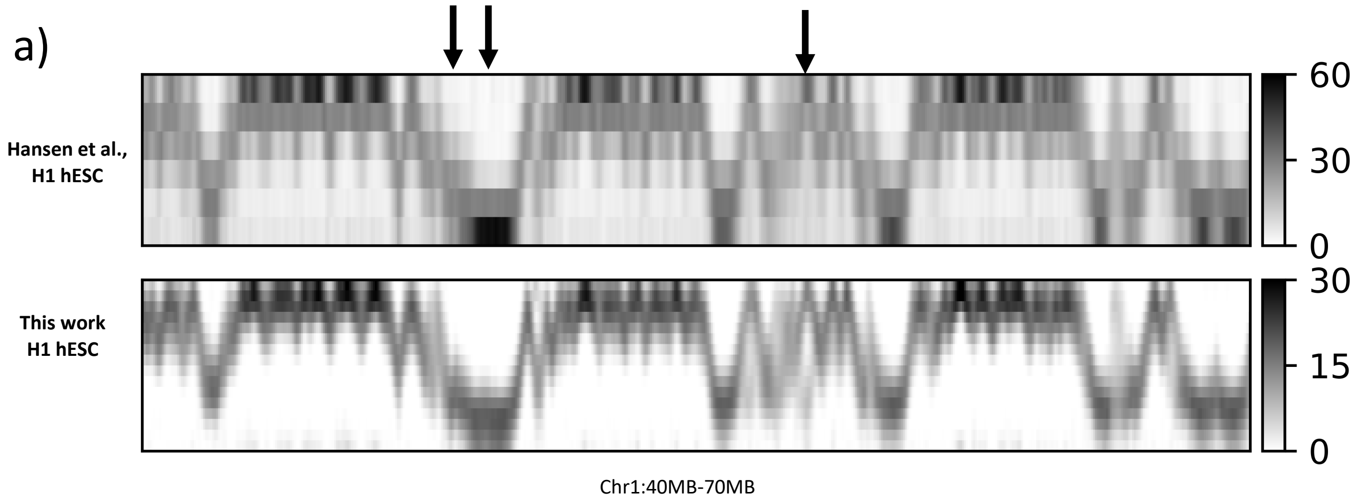
Hansen et al.,
H1 hESC
60
30
0
This work
H1 hESC
30
15
0
Chr1:40MB-70MB

b)

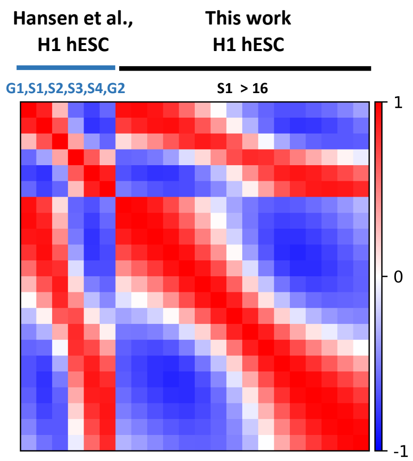
Hansen et al.,
H1 hESC
This work
H1 hESC
G1,S1,S2,S3,S4,G2
S1 > 16
1
0
-1

Fig S8

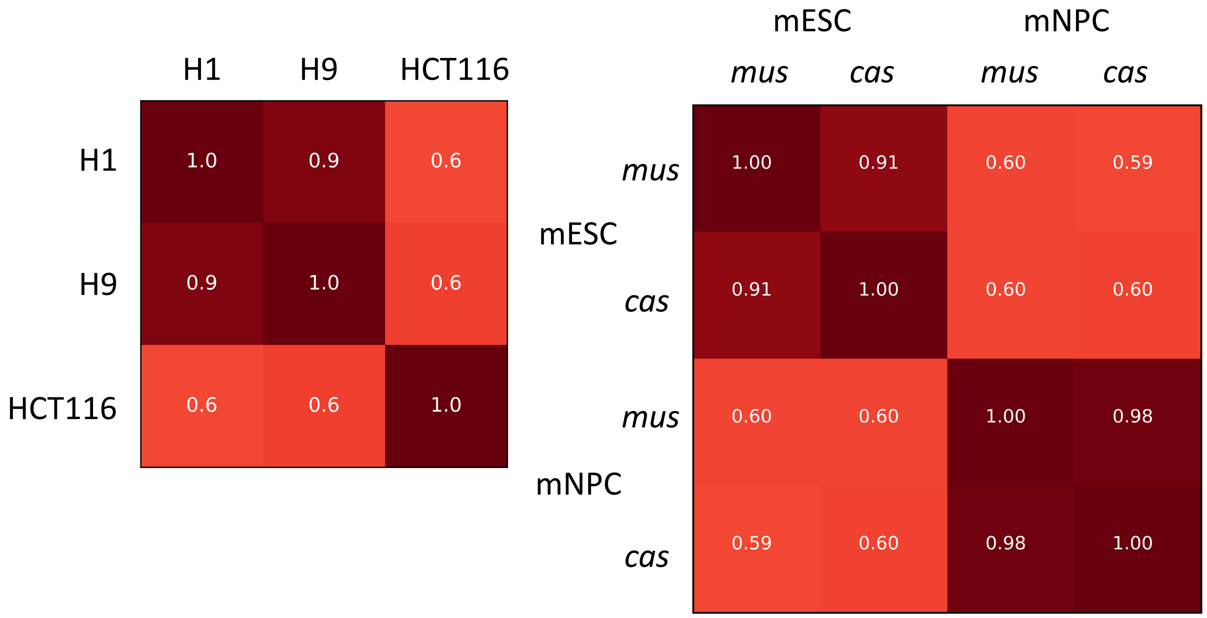

H1
H9
HCT116
H1
H9
HCT116
1.0
0.9
0.6
0.9
1.0
0.6
0.6
0.6
1.0
mESC
mNPC
mus
cas
mus
cas
mESC
mus
cas
mNPC
mus
cas
1.00
0.91
0.60
0.59
0.91
1.00
0.60
0.60
0.60
0.60
1.00
0.98
0.59
0.60
0.98
1.00

Fig S9

a) Sorted BIRCH Centroid

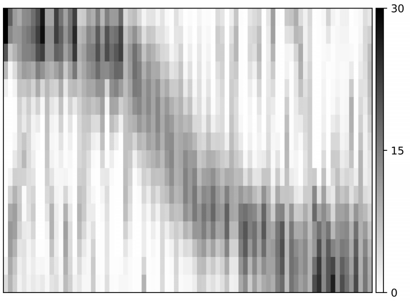

b)

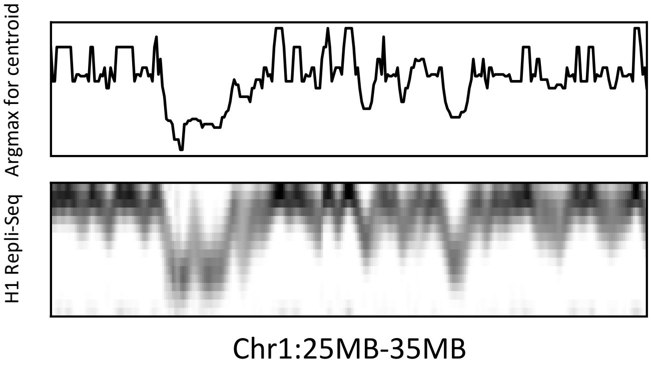

Fig S10

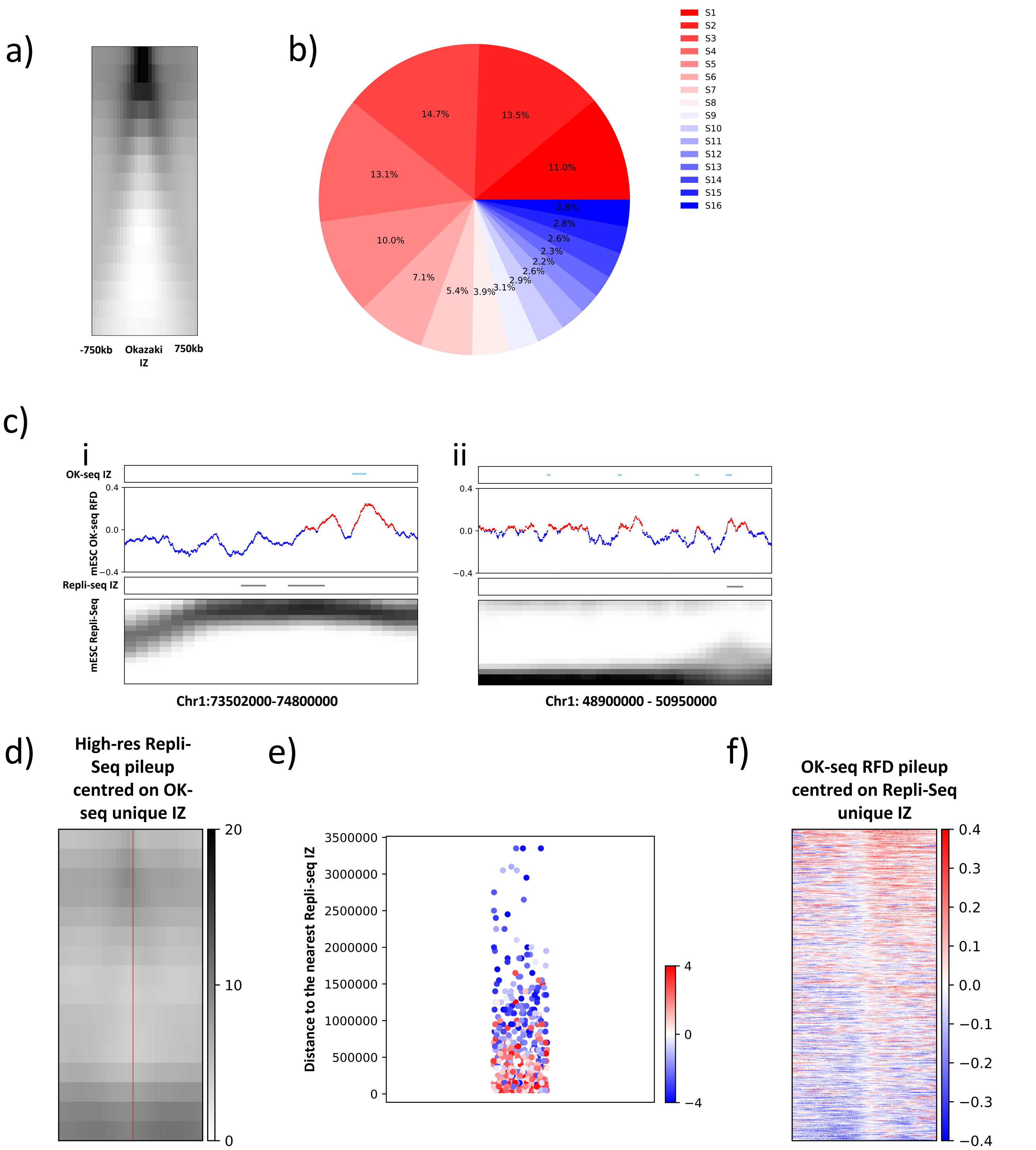
a)
-750kb
Okazaki IZ
750kb
b)
14.7%
13.5%
13.1%
11.0%
10.0%
7.1%
5.4%
3.9%
3.1%
2.9%
2.6%
2.2%
2.3%
2.6%
2.8%
2.8%
S1
S2
S3
S4
S5
S6
S7
S8
S9
S10
S11
S12
S13
S14
S15
S16
c)
i
OK-seq IZ
mESC OK-seq RFD
0.4
0.0
−0.4
Repli-seq IZ
mESC Repli-Seq
Chr1:73502000-74800000
ii
Chr1: 48900000 - 50950000
d)
High-res Repli-Seq pileup centred on OK-seq unique IZ
20
10
0
e)
Distance to the nearest Repli-seq IZ
3500000
3000000
2500000
2000000
1500000
1000000
500000
0
4
0
−4
f)
OK-seq RFD pileup centred on Repli-Seq unique IZ
0.4
0.3
0.2
0.1
0.0
−0.1
−0.2
−0.3
−0.4

Fig S11

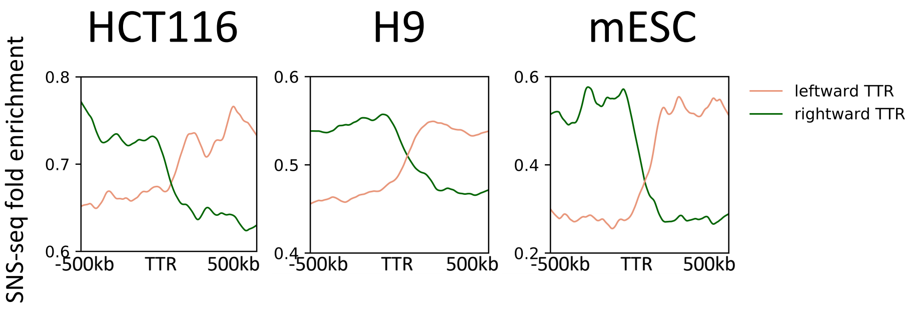
HCT116
H9
mESC
SNS-seq fold enrichment
0.8
0.7
0.6
0.6
0.5
0.4
0.6
0.4
0.2
-500kb
TTR
500kb
leftward TTR
rightward TTR

Fig S12

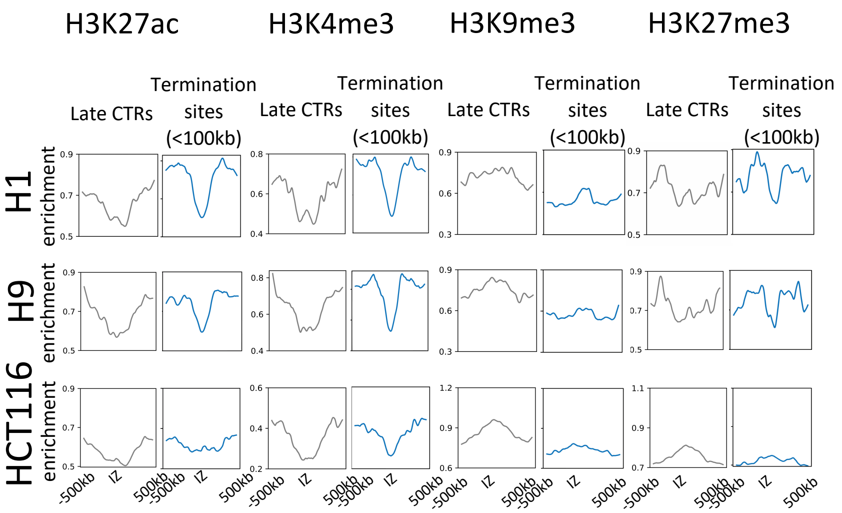

H3K27ac
H3K4me3
H3K9me3
H3K27me3
Late CTRs
Termination sites (<100kb)
H1
H9
HCT116
enrichment
-500kb
IZ
500kb

Fig S13

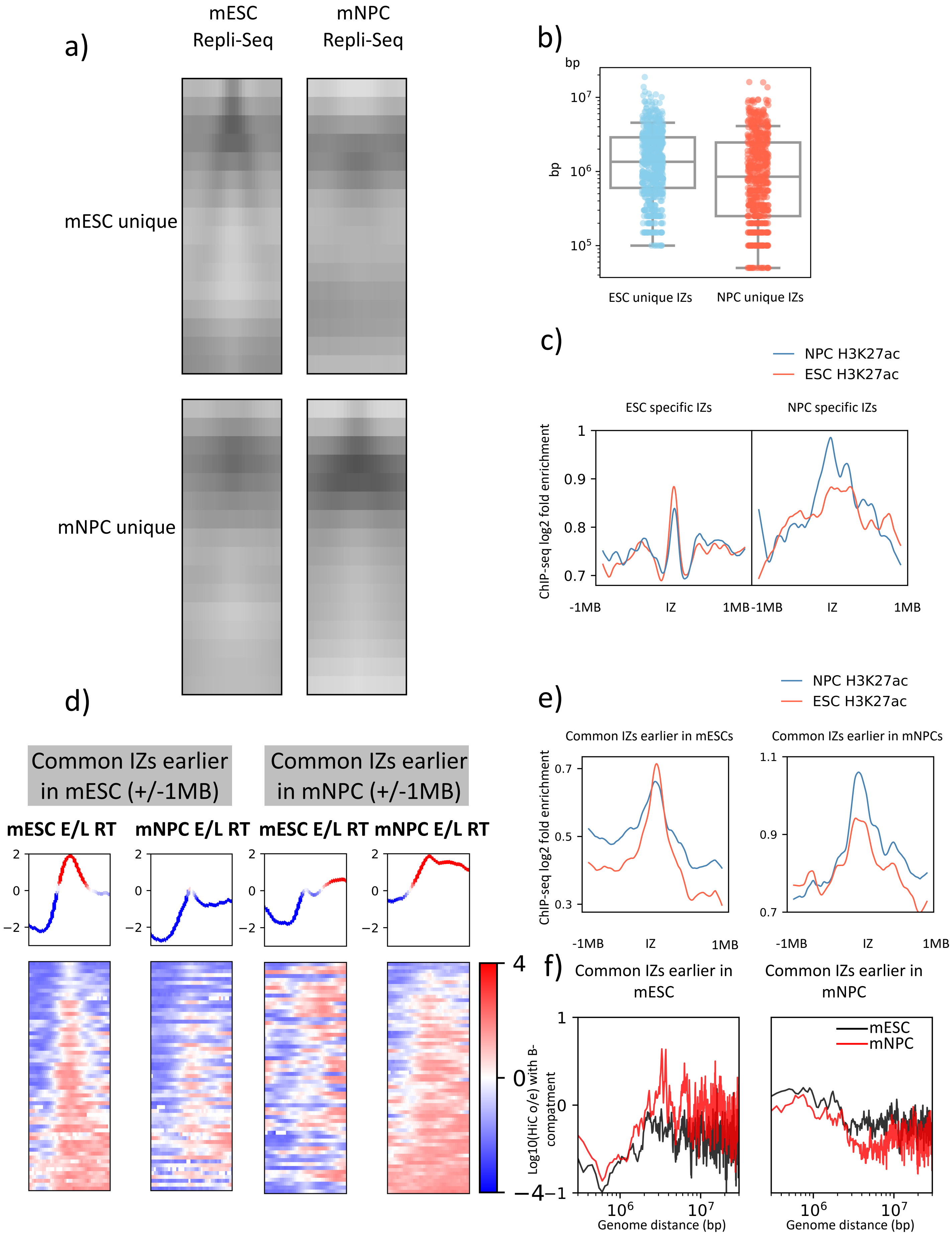

a)
mESC Repli-Seq
mNPC Repli-Seq
mESC unique
mNPC unique
b)
bp
10^7
10^6
10^5
ESC unique IZs
NPC unique IZs
c)
NPC H3K27ac
ESC H3K27ac
ESC specific IZs
NPC specific IZs
ChIP-seq log2 fold enrichment
1
0.9
0.8
0.7
-1MB
IZ
1MB
d)
Common IZs earlier in mESC (+/-1MB)
Common IZs earlier in mNPC (+/-1MB)
mESC E/L RT
mNPC E/L RT
mESC E/L RT
mNPC E/L RT
2
0
−2
4
0
−4
e)
NPC H3K27ac
ESC H3K27ac
Common IZs earlier in mESCs
Common IZs earlier in mNPCs
ChIP-seq log2 fold enrichment
0.7
0.5
0.3
1.1
0.9
0.7
-1MB
IZ
1MB
f)
Common IZs earlier in mESC
Common IZs earlier in mNPC
Log10(HiC o/e) with B-compatment
1
0
−1
mESC
mNPC
10^6
10^7
Genome distance (bp)

Fig S14

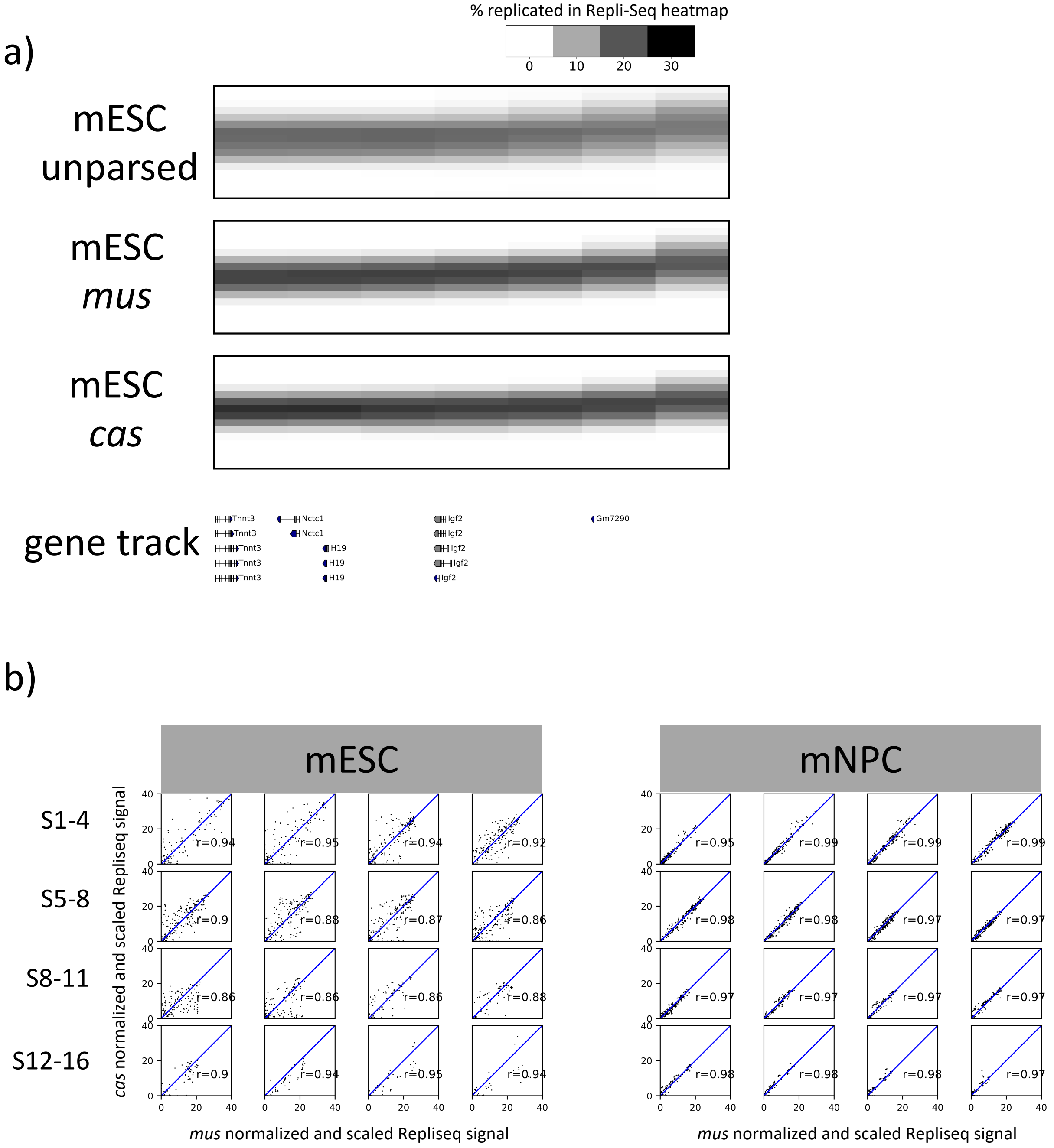
a)
% replicated in Repli-Seq heatmap
0
10
20
30
mESC unparsed
mESC *mus*
mESC *cas*
gene track
Tnnt3
Nctc1
H19
Igf2
Gm7290
b)
mESC
mNPC
S1-4
S5-8
S8-11
S12-16
cas normalized and scaled Repliseq signal
mus normalized and scaled Repliseq signal
r=0.94
r=0.95
r=0.94
r=0.92
r=0.9
r=0.88
r=0.87
r=0.86
r=0.86
r=0.86
r=0.86
r=0.88
r=0.9
r=0.94
r=0.95
r=0.94
r=0.95
r=0.99
r=0.99
r=0.99
r=0.98
r=0.98
r=0.97
r=0.97
r=0.97
r=0.97
r=0.97
r=0.97
r=0.98
r=0.98
r=0.98
r=0.97

Fig S15

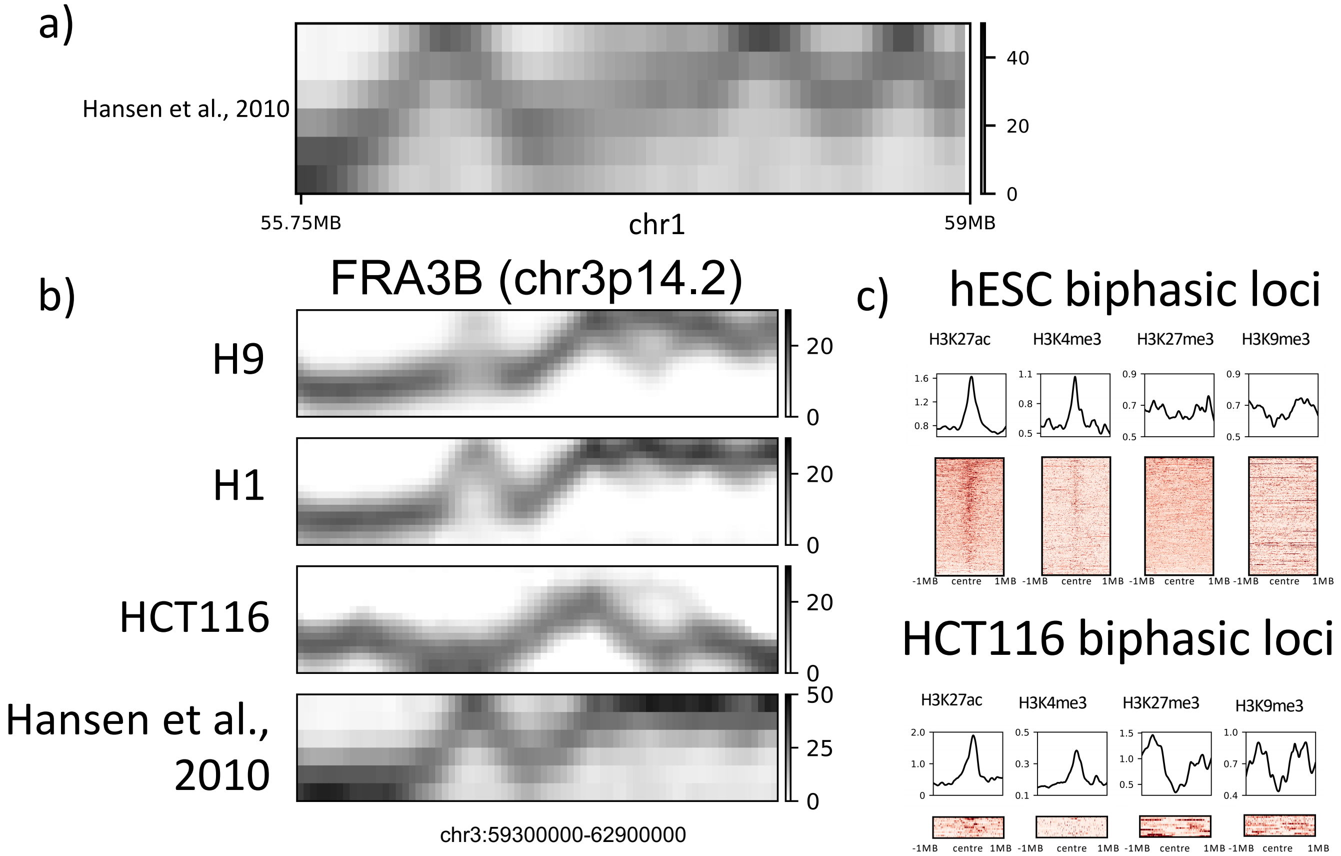
a)
Hansen et al., 2010
40
20
0
55.75MB
chr1
59MB
b)
FRA3B (chr3p14.2)
H9
H1
HCT116
Hansen et al., 2010
20
0
50
25
chr3:59300000-62900000
c)
hESC biphasic loci
H3K27ac
H3K4me3
H3K27me3
H3K9me3
-1MB
centre
1MB
HCT116 biphasic loci

Fig S16

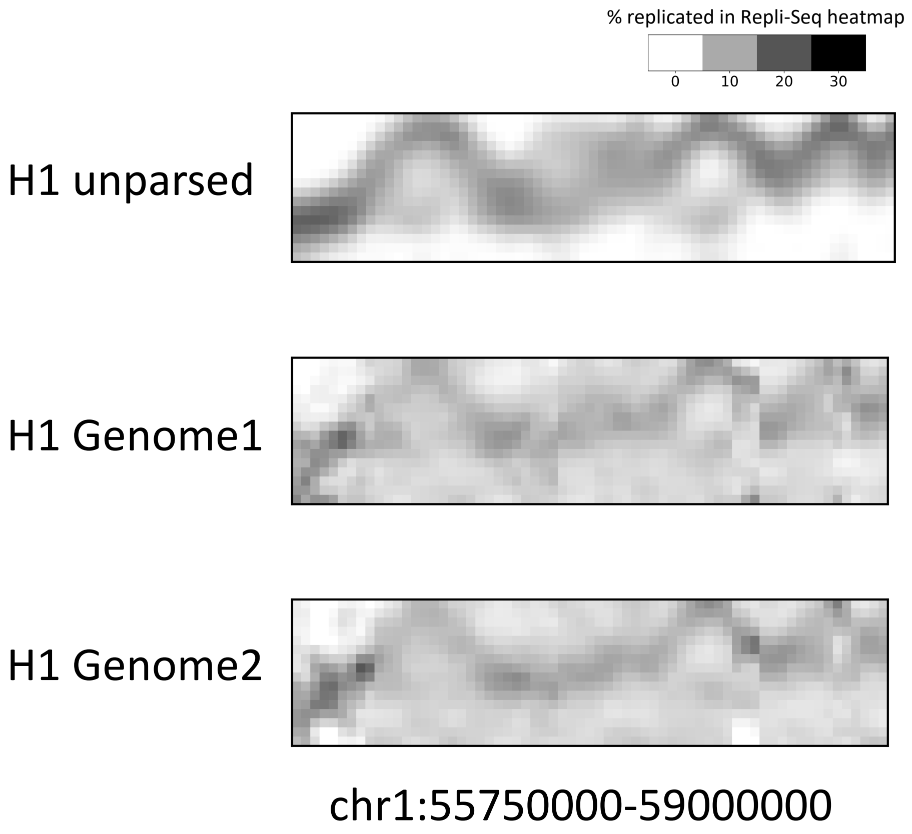
% replicated in Repli-Seq heatmap
0
10
20
30
H1 unparsed
H1 Genome1
H1 Genome2
chr1:55750000-59000000

Supplementary Figure legends

FigureS1 Validation of NPC differentiation by qPCR using primers against Oct4, Dppa2, Nestin, Sox1. The bar graph shows log10 fold enrichment (FE) of the amplicon from corresponding gene product in NPCs relative to that in ESCs. Black lines on bars indicate error bars calculated from triplicates.

FigureS2 Validation of BrdU pull-down by qPCR using primers against alpha- and beta globin. a) Log2 fold enrichment of HBA and HBB in BrdU pull-down DNA from S1-S16 over DNA from G1 for H1, H9, HCT116, mESC and mNPC cell lines. The primer sequences are included in Resource Table II. b) Repli-Seq heatmaps for alpha and beta globin loci in H1, H9, HCT117, mESC and mNPC cell lines with gene tracks for plotted regions at the bottom.

FigureS3 RPM of each S phase fraction is corrected using G1 WGS. a) BrdU pull down efficiency measurement for HCT116 using qPCR. Enrichment was calculated as % spike-in recovery per ng of spike in per ng of input DNA. Black bars indicate enrichment for BrdU labelled spike-in mouse DNA (BrdU pos) and grey bars indicate enrichment for BrdU negative spike-in mouse DNA (BrdU neg) b) Cumulative RPM distribution of each S phase fraction compared with that of G1 in HCT116. c) Cumulative fold enrichment of each S phase fraction after correcting using G1 WGS in HCT116. d) Distribution of log2E/L RT of the bins filtered out, i.e. those that were associated with negative log2 enrichment over G1 WGS, for each S phase fraction of HCT116. Blue and red indicate negative and positive log2E/L RT respectively.

FigureS4 G1 mappability control fraction was devoid of DNA replication. RPM per 50kb bin for H1 G1 cells (top row) and S1 cells (middle row) that have been labelled with BrdU and BrdU immunoprecipitated. Repli-Seq heatmap shown below the tracks. The earliest IZ peaks correspond to the peaks in S1 fraction while G1 control shows a flat profile showing that G1 cells lacked replication and earliest replication was captured in S1.

FigureS5 Normalisation of Repli-Seq heatmaps preserves signal. a) Comparison between heatmap constructed from raw RPM signal and G1 normalised RPM. b) Schematics showing the normalisation of Repli-Seq heatmaps (See Methods). Briefly**,** the heatmap matrix containing the log2 ratio between RPM of BrdU pull-down for S1-S16 and that of G1 WGS was Gaussian smoothed according to equation I (Step I). The smoothed heatmap was scaled column-wise so that all columns add up to 100 to

ensure equal genomic visibility (Step II). The column-wise sums are shown as heatmaps below Repli-Seq heatmaps.

FigureS6 Percentage of replication in S1-S16 for top 10% earliest and latest replicated E/L Repli-Seq bins in mESC, mNPC, H1, H9 and HCT116. The level of noise in normalised and scaled High Resolution Repli-Seq arrays for early replicating bins is assessed by their distribution of percentage of replication in late S phase fractions and vice versa. On average noise accounts for <2% for all cell lines studied.

FigureS7 Comparison between H1 hESC datasets from (9) and H1 hESC datasets from this work a) Example region Chr1:40MB-70MB showing Hansen et al., 2010 H1 datasets Gaussian smoothed and scaled (top row) and H1 hESC datasets from this work (bottom row). b) Spearman's correlation coefficient matrix showing the correlation between S phase fractions of H1 hESC datasets from Hansen et al., 2010 and H1 datasets from this study. Note that the G1 fraction used in Hansen et al., 2010 was the equivalent of the S1 fraction of this study. Our G1 was devoid of nascent DNA (Supplementary Fig4) and used to control for mappability, which was not performed in Hansen et. al.

FigureS8 Correlation heatmaps showing concordance between High-Resolution Repli-Seq datasets of human and mouse cell lines. Spearman's correlation coefficients calculated from comparing 16 concatenated S phase fraction signals from smoothed and scaled Repli-Seq heatmaps of indicated cell lines.

FigureS9 Schematic showing the identification of replication features using BIRCH. a) Cluster centroids sorted according to the S phase fraction where the maximum amount of replication occurs from the earliest to the latest b) Cluster centroid rank profile (top row) and H1 hESC Repli-Seq heatmap (bottom row)

FigureS10 OK-seq IZs are primarily early replicating. a) High-Resolution Repli-Seq pile-up heatmap for called mESC OK-seq IZs. b) Percentage of replication of called mESC OK-seq IZs in each High-Resolution Repli-Seq S phase fraction. c) Example regions where Repli-seq and OK-seq identify different sets of IZs. OK-seq IZs (top first panel) and Repli-Seq IZs (third panel) were indicated with blue bars and grey bars d) Repli-Seq aggregate heatmap centred on mESC OK-seq unique IZs (+/- 1.5MB). The red line indicates the position of OK-seq unique IZs. The aggregate heatmap indicates that Repli-Seq IZs are adjacent to OK-seq unique IZs. Left panel: mESC OK-seq RFD pile-up centred on Repli-Seq unique IZs (+/- 500kb). Right panel: e) Distribution of

distances of ESC OK-seq unique IZs to the nearest Repli-Seq IZ. The points were colour-coded by log2(E/L) RT associated with Repli-Seq IZs.

FigureS11 SNS-seq signal centred around rightward (dark green) and rightward (orange) TTRs in HCT116, H9 and mESCs.

FigureS12 Mean line plots of H3K27ac, H3K4me3, H3K9me3 and H3K27me3 fold enrichment signal centred on late CTRs and termination sites (<100kb) +/- 500kb in HCT116, H1 and H9.

FigureS13 Identification of developmentally regulated IZs in mESC and mNPC. a) mESC (left) and mNPC (right) aggregate Repli-Seq heatmap centred on centres of mESC unique IZs (top) and mNPC unique IZs (top) +/- 750kb. b) Boxplots showing the distribution of distances of mESC- and mNPC-unique IZs from the nearest mESC- and mNPC-unique IZs respectively. c) mNPC (pink) and mESCs (blue) H3K27ac ChIP-Seq log2 fold enrichment signal +/- 1MB around IZs that are mESC unique (left panel) or mNPC unique (right panel). d) mESC and mNPC Log2E/L RT centred at common IZs initiating at significantly different times in mESCs and mNPCs (earlier in mESCs: left panel, earlier in mNPCs: right panel) +/- 1MB. Line plots were colour-coded with blue and red representing negative and positive log2E/L RT in the corresponding cell line. e) mNPC (pink) and mESCs (blue) H3K27ac ChIP-Seq log2 fold enrichment signal +/- 1MB around IZs that are earlier replicating in mESCs (left panel) or in mNPCs (right panel). f) Lineplots showing Log10 observed over expected (o/e) HiC interactions with B-compartment in mESC (black) and mNPC (red) for IZs that are earlier replicating in mESCs (left) and mNPC (right) against genomic distance (bp) on a log scale.

FigureS14 Imprinted genes do not exhibit biphasic patterns. a) mESC unparsed and allele-parsed Repli-Seq heatmaps showing an example imprinted locus (H19-Igf2) in mESC (chr7:142500000-142850000). b) Scatter plots showing the correlation between normalised read densities on *mus* and *cas* alleles in High-Resolution Repli-Seq heatmaps at imprinted loci for fractions S1-S16 in mESC (left) and mNPC (right)**.**

FigureS15 Biphasic sites overlap with CFSs and are enriched for active histone marks. a) FRA1B (same locus as shown in **Fig7a** in H1 hESC datasets from (9) b) Repli-Seq profiles of FRA3B (chr3p14.2) in H9, H1 and HCT116 cells from this study and H1 hESC from (9). c) H3K27ac, H3K4me3, H3K9me3 and H3K27me3 ChIP-seq fold enrichment signal centred on biphasic loci in H9 and HCT116 cell lines.

FigureS16 H1 hESC unparsed and allele-parsed Repli-Seq heatmaps for chr1:55,750,000 – 59,000,000, the locus shown in Fig6a.
