# Supplementary material for: High-resolution Repli-Seq defines the temporal choreography of initiation, elongation and termination of replication in mammalian cells
Source: Genome Biol. 2020 Mar 24;21:76. doi: 10.1186/s13059-020-01983-8 (PMC7092589; doi:10.1186/s13059-020-01983-8)
Supplement: Supplementary file 2 — Additional file 2 : Table S1 Primer lists for human and mouse. Table S2 Resource table stating sources of external datasets. [file 13059_2020_1983_MOESM2_ESM.docx]

Table S1

| **Mouse** |  |
| --- | --- |
| **Primer name** | **Sequences (5’-3’)** |
| GAPDH_F | TTGATGGCAACAATCTCCAC |
| GAPDH_R | CGTCCCGTAGACAAAATGGT |
| Oct4_F | TCTTCTGCTTCAGCAGCTTG |
| Oct4_R | GTTGGAGAAGGTGGAACCAA |
| Dppa2_F | AGCATGCCTCACCAGAGAAC |
| Dppa2_R | CCCTTTAAGGAGGAGGAGGA |
| Nestin_F | GTCAGATCGCTCAGATCCT |
| Nestin_R | AGCAGAGTCCTGTATGTAGC |
| Sox1_F | TGTAATCCGGGTGTTCCTTC |
| Sox1_R | TGTAATCCGGGTGTTCCTTC |
| \| mHBA_F \|  \| \| --- \| --- \| | GCATCTGGGACCCCTAGGAA |
| mHBA_R | CGGAGACAAAGTGGACACCC |
| mHBB_F | TTCCAACTGGGGAAAAGGCTC |
| mHBB_R | TTGGTGCTATCTTTGCATGGTA |
| Mito(labelled)_F | GACATCTGGTTCTTACTTCA |
| Mito(labelled)_R | GTTTTTGGGGTTTGGCATTA |
| Dppa(unlabelled)_F | CCACAGGAAGACAGGAAGCAGT |
| Dppa(unlabelled)_R | AGCCAGACAGGAGCCCTAGAGT |
| **Human** |  |
| **Primer name** | **Sequences (5’-3’)** |
| hHBA_F | GCCTCCCTGGACAAGTTCCT |
| hHBA_R | CCGAGGCTCCAGCTTAACG |
| hHBB_F | GGGGATCTGTCCACTCCTGA |
| hHBB_R | CCAGGCCATCACTAAAGGCA |

Table S2

| RESOURCE | SOURCE | IDENTIFIER |
| --- | --- | --- |
| Deposited Data |  |  |
| H9 H3K27me3 ChIP-seq | Lyu et al., 2018 | GEO: GSM2816652 |
| H9 H3K27ac ChIP-seq | Lyu et al., 2018 | GEO: GSM2816648 |
| H9 H3K4me3 ChIP-seq | Lyu et al., 2018 | GEO: GSM2816656 |
| H9 H3K9me3 ChIP-seq | UCSD Human Reference Epigenome Mapping Project | GEO: GSM667631 |
| H9 RNA-seq | Nguyen et al., 2018 | GEO: GSM2690858 |
| H1 H3K27me3 Cut&Run | Janssens et al., 2018 | GEO: GSM3391653 |
| H1 H3K27ac Cut&Run | Janssens et al., 2018 | GEO: GSM3391652 |
| H1 H3K4me3 Cut&Run | Janssens et al., 2018 | GEO: GSM3391656 |
| H1 H3K9me3 ChIP-seq | ENCODE Project Consortium, 2012 | GEO: GSM1003585 |
| H1 RNA-seq | UCSD Human Reference Epigenome Mapping Project | GEO:GSM438361 |
| mESC H3K27me3 ChIP-seq | ENCODE Project Consortium, 2012 | GEO: GSM1000089 |
| mESC H3K27ac ChIP-seq | ENCODE Project Consortium, 2012 | GEO: GSM1000126 |
| mESC H3K4me3 ChIP-seq | ENCODE Project Consortium, 2012 | GEO: GSM1000124 |
| mESC H3K9me3 ChIP-seq | ENCODE Project Consortium, 2012 | GEO: GSM1003751 |
| mESC RNA-seq | Rivera-Mulia et al., 2018 | **GEO: GSE07421** |
| mNPC H3K27me3 ChIP-seq | Liu et al., 2017 | GEO: GSM2442461 |
| mNPC H3K27ac ChIP-seq | Wang et al.,2018 | GEO: GSM2805446 |
| mNPC H3K4me3 ChIP-seq | Liu et al., 2017 | GEO: GSM2442457 |
| mNPC H3K9me3 ChIP-seq | Bulut-Karslioglu et al.,2014 | GEO: GSM1375164 |
| H9 Hi-C | Lyu et al., 2018 | GEO: GSM2816607 |
| HCT116 Hi-C | Rao et al., 2017 | GEO: GSM2795535 |
| H1 Hi-C | Dekker Lab for 4DN Project Consortium | Unpublished, 4DN accession number: 4DNESRJ8KV4Q |
| mESC Hi-C | Bonev et al., 2017 | GEO: GSE96107 |
| mNPC Hi-C | Bonev et al., 2017 | GEO: GSE96107 |
| mESC SNS-seq | Cayrou et al., 2015 | GEO: GSE68347 |
| mESC OK-seq | Petryk et al., 2018 | GEO: GSM3290342 |
| H9 SNS-seq | Bersnard et al.,2012 | GEO: GSM927236 |
| HCT116 SNS-seq | Fu et al., 2015 | GEO: GSM1648749 |
| Raw and analysed data | This paper | GEO: GSE137764 |
